# Recombinant production of the lantibiotic nisin using *Corynebacterium glutamicum* in a two-step process

**DOI:** 10.1186/s12934-022-01739-y

**Published:** 2022-01-15

**Authors:** Dominik Weixler, Max Berghoff, Kirill V. Ovchinnikov, Sebastian Reich, Oliver Goldbeck, Gerd M. Seibold, Christoph Wittmann, Nadav S. Bar, Bernhard J. Eikmanns, Dzung B. Diep, Christian U. Riedel

**Affiliations:** 1 Institute of Microbiology and Biotechnology, University of Ulm, Albert-Einstein-Allee 11, 89081 Ulm, Germany; 2 Faculty of Chemistry, Biotechnology and Food Science, Norwegian University of Life Sciences, Ås, Norway; 3 Department of Biotechnology and Biomedicine, Technical University of Denmark, Lyngby, Denmark; 4 Institute of Systems Biotechnology, Saarland University, Saarbrücken, Germany; 5 Department of Chemical Engineering, Norwegian University of Science and Technology, Trondheim, Norway

**Keywords:** *Corynebacterium glutamicum*, Bacteriocin, Nisin, Recombinant production, Pre-nisin, Biosensor

## Abstract

**Background:**

The bacteriocin nisin is naturally produced by *Lactococcus lactis* as an inactive prepeptide that is modified posttranslationally resulting in five (methyl-)lanthionine rings characteristic for class Ia bacteriocins. Export and proteolytic cleavage of the leader peptide results in release of active nisin. By targeting the universal peptidoglycan precursor lipid II, nisin has a broad target spectrum including important human pathogens such as *Listeria monocytogenes* and methicillin-resistant *Staphylococcus aureus* strains. Industrial nisin production is currently performed using natural producer strains resulting in rather low product purity and limiting its application to preservation of dairy food products.

**Results:**

We established heterologous nisin production using the biotechnological workhorse organism *Corynebacterium glutamicum* in a two-step process. We demonstrate successful biosynthesis and export of fully modified prenisin and its activation to mature nisin by a purified, soluble variant of the nisin protease NisP (sNisP) produced in *Escherichia coli*. Active nisin was detected by a *L. lactis* sensor strain with strictly nisin-dependent expression of the fluorescent protein mCherry. Following activation by sNisP, supernatants of the recombinant *C. glutamicum* producer strain cultivated in standard batch fermentations contained at least 1.25 mg/l active nisin.

**Conclusions:**

We demonstrate successful implementation of a two-step process for recombinant production of active nisin with *C. glutamicum*. This extends the spectrum of bioactive compounds that may be produced using *C. glutamicum* to a bacteriocin harboring complex posttranslational modifications. Our results provide a basis for further studies to optimize product yields, transfer production to sustainable substrates and purification of pharmaceutical grade nisin.

**Supplementary Information:**

The online version contains supplementary material available at 10.1186/s12934-022-01739-y.

## Background

Massive over- and misuse of antibiotics in medicine and animal farming for food production has fueled the development of antibiotic resistance of a wide range of pathogens [1–3]. In 2014, the global increase in antibiotic resistant bacteria has been recognized by the World Health Organization (WHO) as one of the most urgent problems to human health. Consequently, the WHO has issued a warning of a post-antibiotic era in which infections with common bacteria become lethal are a realistic scenario [4]. Replacement of antibiotics with bacteriocins and other antimicrobial peptides may contribute to solve the problems of antibiotic resistance.

Bacteriocins are a group of ribosomally synthesized peptides produced by different bacteria that show high antimicrobial activity against various bacteria including antibiotic resistant human pathogens [5, 6]. Thus, bacteriocins may be an interesting alternative to classic antibiotics [5, 7]. One of the best characterized bacteriocins is the lanthipeptide nisin, which is naturally produced by different *L. lactis* strains [8]. There are several natural variants of nisin (e.g. nisin A, Z, and Q) with slightly different amino acid sequences but conserved secondary structure. The mature peptides consist of 34 amino acids with multiple posttranslational modifications including dehydrated amino acids and formation of so-called (methyl-)lanthionine rings [8].

The enzymes for nisin biosynthesis are encoded by a cluster of genes for the prepeptide (dependent on the nisin variant *nisZ*, *nisA*, etc.), modification (*nisB* and *nisC*), regulation (*nisRK*), transport (*nisT*) and immunity (*nisI; nisFEG*) [8–10]. The *nisA*- or *nisZ*-encoded precursor peptide (“prenisin”) consists of 57 amino acids and is subsequently processed and exported by the enzymes NisB, NisC, NisT and NisP [8]. NisB is a dehydratase that converts threonine and serine residues to the dehydroamino acids dehydroalanine and dehydrobutyrine, respectively [11, 12]. In a second modification step, the cyclase NisC couples the dehydroamino acids to specific cysteine residues by formation of thioether bonds resulting in five characteristic (methyl-)lanthionine rings [13, 14]. The fully modified but still inactive prenisin is then transported to the extracellular space via the ABC-transporter NisT [15–17]. Finally, proteolytic cleavage of the leader peptide between arginine and isoleucine residues in position 23–24 by the membrane anchored protease NisP leads to activation and release of mature and active nisin [18–20]. This final proteolytic activation by NisP requires a fully modified prenisin [21, 22].

To achieve growth inhibition and killing of bacteria, nisin targets the bacterial cell wall precursor lipid II [23, 24] located in the outer leaflet of the cell membrane by binding to the pyrophosphate unit of lipid II [25]. This leads to inhibition of growth by sequestering lipid II and preventing its incorporation into the nascent peptidoglycan chain at the cell septum. With increasing concentration of nisin at the membrane, the bactericidal activity of nisin is mediated by pore-forming complexes consisting of eight molecules of nisin and four molecules of lipid II [23, 26]. Nisin exhibits antimicrobial activity against a wide spectrum of Gram-positive bacteria including important human pathogens such as *Listeria monocytogenes*, *Enterococcus* sp., *Staphylococcus aureus* strains [27–32]. Due to its broad spectrum of target organisms and classification as a generally regarded as safe substance, nisin is authorized as food additive by the European Food Safety Authority (ESFA; E number: E234) and U.S. Food and Drug Authority (FDA) and widely used in food preservation [33]. The global market volume of nisin is steadily increasing and projected to exceed 500 Mio. USD in 2025 (https://www.marketsandmarkets.com/Market-Reports/nisin-market-29041412.html). Due to its activity against antibiotic resistant strains of *Enterococcus* sp. and *S. aureus* [29–32] it is also discussed as an alternative to treat infections with these organisms [5].

So far, industrial scale production of nisin is performed exclusively with natural *L. lactis* producer strains [34]. These production processes bear several disadvantages including expensive media and intensive downstream processing [34–36]. In consequence, nisin preparations are sold as partial purified product containing only 2.5% active nisin [34, 37]. Hence, recombinant production of nisin using a robust biotechnological workhorse may increase product yields and improve product purity by using defined media and well-established fermentation and down-stream processes. The Gram-positive bacterium *Corynebacterium glutamicum* is a well-established host for a wide range of compounds including high value active ingredients, therapeutic proteins and supplements for medial infusion solutions [38–44]. Recently, we successfully established *C. glutamicum* as a host to produce the bacteriocin pediocin PA-1, a class IIa bacteriocin that is not extensively modified [45]. With the present study, we extend the range of antimicrobial peptides that can be produced using *C. glutamicum* by implementing a two-step process for production of the completely modified class I bacteriocin nisin.

## Results

### *C. glutamicum* is not suitable for production of active nisin

Although previous studies reported *C. glutamicum* to be resistant to up to 40 µg/ml of nisin [46], our own results suggest that growth of *C. glutamicum* is completely inhibited by ~ 1 µg/ml of nisin [47, 48]. To corroborate these results and to determine the nisin concentration that is required for formation of pores in the membrane of *C. glutamicum*, we devised a biosensor expressing the pH-dependent fluorescent protein pHluorin2 [49] in a similar fashion as described recently for *L. monocytogenes* [50]. For this biosensor, a *pHluorin2* gene sequence codon-optimized for *C. glutamicum* was fused to the strong, constitutive *tuf* promoter [51], cloned into the pPBEx2 plasmid [52] and introduced into *C. glutamicum* ATCC 13032 (Additional file 1: Fig. S1A, B).

**Fig. 1:**
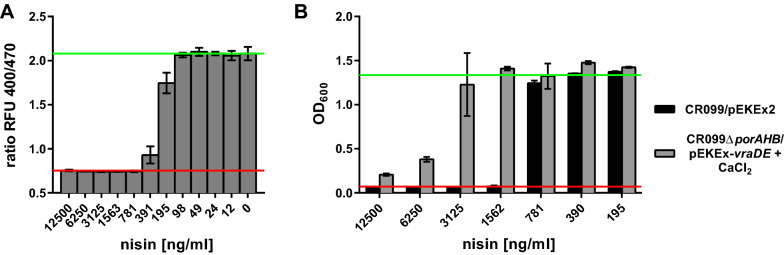
Sensitivity of *C. glutamicum* to nisin. **A** Membrane damage of *C. glutamicum* CR099/pPB-pHin2^*Cg*^ biosensor in the presence of different concentrations of nisin. Values are ratios of fluorescence intensity with emission at 510 nm after excitation at 400 and 470 nm (ratio RFU 400/470). **B** Optical density (OD_600_) of *C. glutamicum* CR099/pEKEx2 and CR099Δ*porA*Δ*porH*Δ*porB/*pEKEx-*vraDE* after 24 h of incubation in the presence of nisin at the indicated concentrations (195–12,500 ng/ml). For assays bacteria were harvested from fresh o/N cultures, resuspended in fresh medium at an OD_600_ of 0.05 in 2xTY medium. For CR099Δ*porA*Δ*porH*Δ*porB/*pEKEx-*vraDE*, medium was additionally supplemented with 2 g/l CaCl_2_. All values are mean ± standard deviation (SD) of triplicate measurements of one representative of three independent cultures per strain. The red and green lines indicate OD_600_ or ratio RFU 400/470 of the positive (i.e. complete inhibition of growth) or negative (i.e. in the absence of nisin) control, respectively

*Corynebacterium glutamicum* ATCC 13032/pPB-pHin^*Cg*^ shows high fluorescence with two excitation maxima and the characteristic ratiometric, pH-dependent shift following cetyltrimethylammoniumbromid (CTAB)-induced disturbance of membrane integrity (Additional file 1: Fig. S1C, D). Exposure of this strain to a range of nisin concentrations revealed that as little as 195 ng/ml are sufficient to elicit signs of membrane damage and at concentrations above 781 ng/ml membrane integrity of all bacteria was completely disrupted (Fig. 1A). This indicates that recombinant production of active nisin is not possible using *C. glutamicum* as the product not only inhibits growth [47, 48] but kills producer cells at low concentrations.

Recently, we tested several approaches to improve resistance of *C. glutamicum* to nisin by e.g. expression of nisin immunity genes, ABC-transporters of pathogenic bacteria known to confer protection against nisin and their homologs of *C. glutamicum*, deletion of porins, or modification of cell surface charge [47]. Some of these approaches yielded marginal improvements but, individually, none of them increased resistance more than two-fold. To test if they would synergize to create a more resistant strain suitable for production of active nisin, we combined several of these approaches creating *C. glutamicum* CR099Δ*porA*Δ*porH*Δ*porB/*pEKEx-*vraDE*. This strain carries clean deletions of the genes for porins PorA, PorH and PorB and harbours a plasmid for expression of the VraDE ABC-transporter of *S. aureus*, which is known to confer nisin resistance in this organism [53, 54]. When cultivated in the presence of 2 g/l CaCl_2_, i.e. a condition that also slightly increases resistance of *C. glutamicum* to nisin [47], this strain only showed about eightfold higher resistance to (Fig. 1B). Nevertheless, growth of *C. glutamicum* CR099Δ*porA*Δ*porH*Δ*porB/*pEKEx-*vraDE* under these conditions was also markedly reduced at concentrations around 5 µg/ml. Due to the rather low resistance to nisin and the observed pore formation at concentrations of ~ 200 ng/ml, we concluded that *C. glutamicum* is not suitable as a recombinant host for production of active nisin.

### Construction of a nisin-specific biosensor

To be able to detect nisin in a specific manner, we first established a whole-cell biosensor based on nisin-inducible expression [55, 56] of a fluorescent protein in a *L. lactis* host. The promoter upstream of *nisZ* (P_*nis*_) of *L. lactis* B1629 was amplified and fused to the gene for the fluorescent protein mCherry, which was optimized for codon usage of *L. lactis* (*mcherry*^*Ll*^). This construct was cloned into the pNZ44 backbone and the obtained plasmid pNZ-P_*nis*_*-mcherry*^*Ll*^ (Additional file 1: Fig. S2A) was introduced into *L. lactis* NZ9000 harbouring the genes coding for the two component nisin regulation system *nisK* (sensor kinase) and *nisR* (regulator) [56].

Following growth o/N in the presence of 10 ng/ml nisin, pellets of *L. lactis* NZ9000/ pNZ-P_*nis*_*-mcherry*^*Ll*^ had a reddish colour indicating efficient expression of mCherry (Additional file 1: Fig. S2B). Further dose response experiments revealed a limit of detection of 0.1–0.2 ng/ml, maximum expression at 1.5–2 ng/ml (data not shown), and a linear dose response between 0 and 1 ng/ml (Additional file 1: Fig. S2C).

### Production of prenisin using *C. glutamicum* and activation by trypsin

Based on the high sensitivity of *C. glutamicum* towards nisin and the unsuccessful attempts to create recombinant strains with significantly improved resistance we sought to establish a two-step process with production of (inactive) prenisin and downstream activation to (active) nisin by trypsin treatment as described previously [21, 22, 57]. To produce prenisin using *C. glutamicum*, the genes *nisZ, nisB, nisT* and *nisC* of the nisin Z biosynthesis operon of *L. lactis* B1629 (Additional file 1: Fig. S3A) were obtained as synthetic DNA fragments optimized for codon usage of *C. glutamicum* each equipped with a ribosomal binding site. The synthetic *nisZBTC*^*Cg*^ operon was cloned downstream of the P_tac_ promoter into pXMJ19 (Additional file 1: Fig. S3B) and the obtained plasmid pXMJ-*nisZBTC*^*Cg*^ was introduced into *C. glutamicum* CR099.

**Fig. 2:**
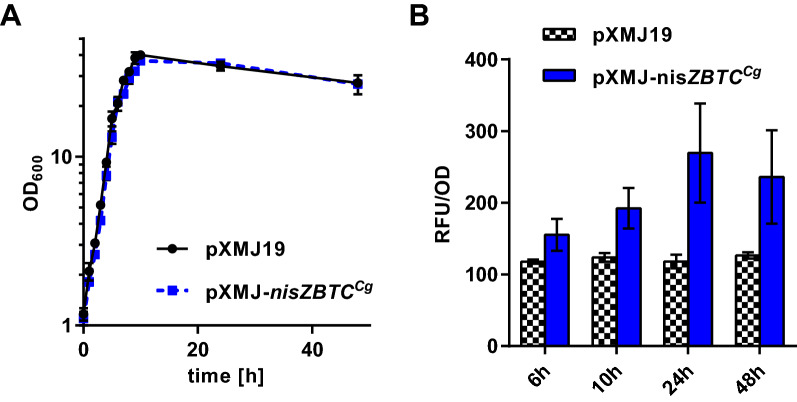
Production of prenisin using *C. glutamicum* and activation of prenisin to nisin culture supernatants. **A** Growth of *C. glutamicum* CR099/pXMJ-*nisZBTC*^*Cg*^ and CR099/pXMJ19 on 2xTY medium with 2% glucose and induction with IPTG (0.2 mM) after 2 h of growth. **C** Relative mCherry fluorescence normalized to OD_600_ (RFU/OD) of *L. lactis* NZ9000/pNZ-P_*nis*_*-mcherry*^*Ll*^ grown o/N in the presence of supernatants of *C. glutamicum* CR099/pXMJ-*nisZBT*^*Cg*^ and CR099/pXMJ19 harvested at the indicated time points of the experiment shown in (**B**). Prior to assays, supernatants were activated by incubation with trypsin (0.5 mg/ml for 3.5 h) and diluted 1:100. Values are mean ± SD of n = 3 independent cultures

Growth of *C. glutamicum* CR099/pXMJ-*nisZBTC*^*Cg*^ on 2xTY complex medium containing 2% (w/v) glucose with addition of IPTG after 2 h was comparable to that of the empty vector control strain *C. glutamicum* CR099/pXMJ19 (Fig. 2A). Of note, supernatants of *C. glutamicum* CR099/pXMJ-*nisZBTC*^*Cg*^ were able to induce fluorescence in the nisin biosensor strain *L. lactis* NZ9000/pNZ-P_*nis*_*-mcherry*^*Ll*^ following trypsin activation and fluorescence per OD increased with time (Fig. 2B). By contrast, supernatants of the empty vector control strain treated with trypsin did not induce fluorescence of the biosensor above background.

**Fig. 3:**
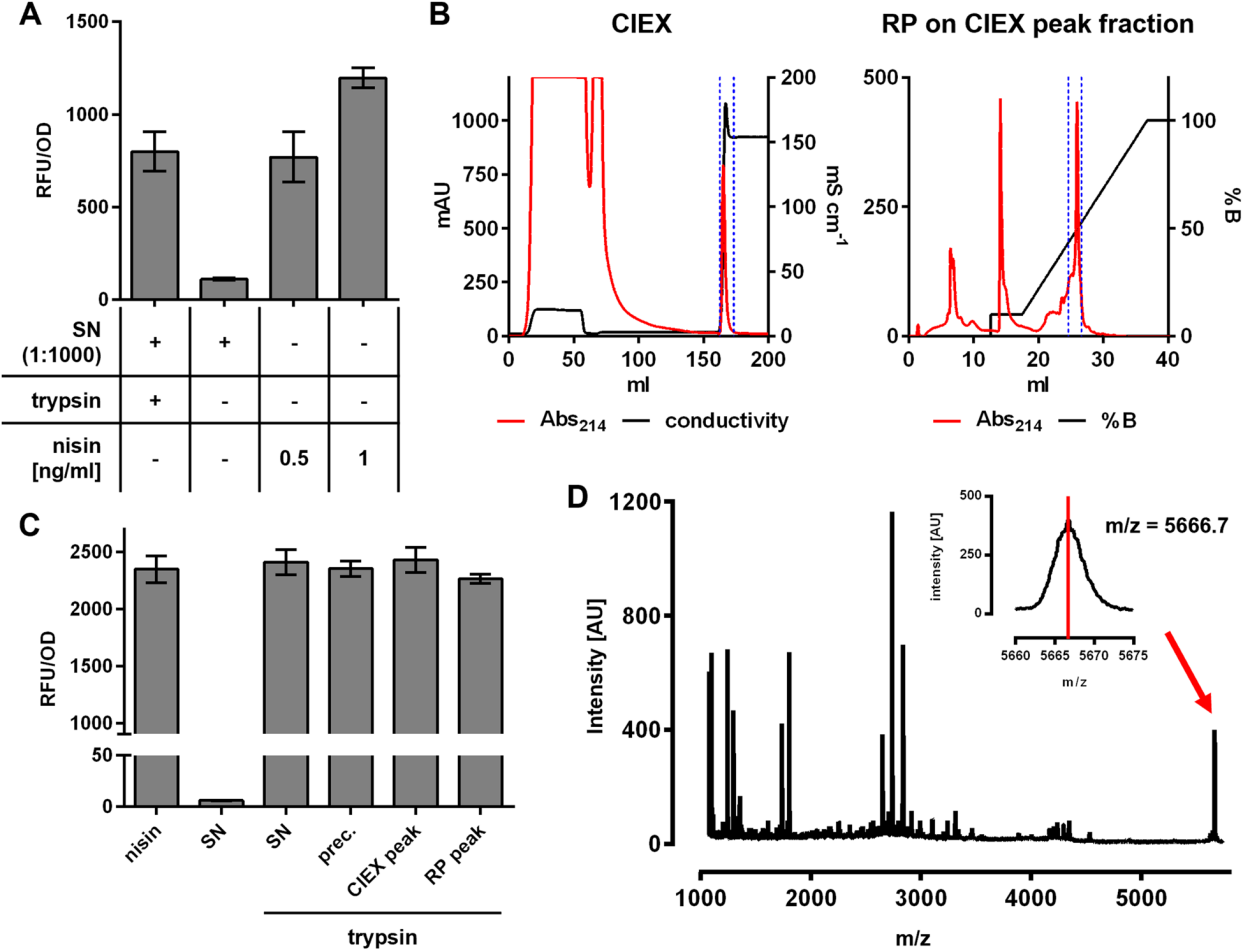
Purification and activation of prenisin produced by *C. glutamicum*. **A** Relative mCherry fluorescence normalized to OD (RFU/OD) of *L. lactis* NZ9000/pNZ-P_*nis*_*-mcherry*^*Ll*^ grown o/N in the presence of supernatants (SN) of *C. glutamicum* CR099/pXMJ-*nisZBTC*^*Cg*^. The producer was grown o/N in 2xTY with 2% Glc and 0.2 mM IPTG. **B** Purification of ammonium sulphate-precipitated SN proteins by cation exchange (CIEX) and subsequent reverse phase (RP) chromatography on the CIEX peak fraction. Indicated is absorbance at 214 nm (red) and conductance (mS/cm; black, in CIEX) or % of elution buffer (%B, black, in RP) over the elution volume. Boundaries of the peak fractions further analysed are marked with blue broken lines. **C** RFU/OD of *L. lactis* NZ9000/pNZ-P_*nis*_-*mcherry*^*Ll*^ grown o/N in the presence of samples obtained at different steps during the purification of prenisin from SN shown in (**A**). prec: ammonium sulphate-precipitated SN proteins resuspended in pure H_2_O; CIEX and RP peak: peak fraction of the CIEX and RP chromatography. Prior to assays, samples were activated by incubation with trypsin (0.5 mg/ml for 3.5 h) and diluted 1:1000. As positive controls, the biosensor was grown in the presence of nisin Z at the indicated concentration. As negative controls, SN without trypsin treatment were included. **D** Mass spectrometry of the peak fraction obtained in RP chromatography in (**B**) with arbitrary peak intensity units (intensity [AU]) over mass/charge ratio (m/z). Values in (**A**) and (**C**) are mean ± SD of n = 3 independent cultures (**A**) or technical triplicates of one representative preparation (**C**)

To further identify and characterize the compound produced by *C. glutamicum* CR099/pXMJ-*nisZBTC*^*Cg*^, supernatant proteins were precipitated and analyzed by chromatography and mass spectrometry. Conditions of cultivation were slightly different with both precultures and main cultures supplemented with IPTG and supernatants harvested after o/N growth. Under these conditions, fluorescence of the biosensor incubated with supernatants diluted 1:1000 after trypsin activation was comparable to biosensors incubated in the presence of 0.5 ng/ml of nisin (Fig. 3A). This suggests the trypsin-treated supernatants of *C. glutamicum* CR099/pXMJ-*nisZBTC*^*Cg*^ contain at least 0.5 µg/ml of active nisin. Supernatants of these cultivations were harvested, and proteins were precipitated and purified by chromatography. In cation exchange (CIEX) chromatography a single peak in absorbance at 214 nm that coincided with a steep increase in conductivity was observed at the onset of elution (Fig. 3B). The peak fraction was collected and further analyzed by reverse phase (RP) chromatography with a two-step elution profile. This yielded a sharp peak in the second elution step at around 50% acetonitrile (Fig. 3B).

Subsequent analysis of precipitated supernatant proteins, CIEX and RP peak fractions suggests that all samples contained a compound that induced fluorescence by the nisin-specific biosensor following activation by trypsin (Fig. 3C). Moreover, a signal with a mass/charge ratio (m/z) of 5666.7 was detected in MALDI-TOF/MS analyses (Fig. 3D). This is close to the predicted m/z of the nisin Z prepeptide harboring all required posttranslational modifications with a neutral net charge (Additional file 1: Fig. S3C). Collectively, these results suggest that recombinant production of fully modified prenisin using *C. glutamicum* and a protocol for purification and down-stream activation to nisin was established.

### Improved activation of prenisin by recombinant NisP

**Fig. 4:**
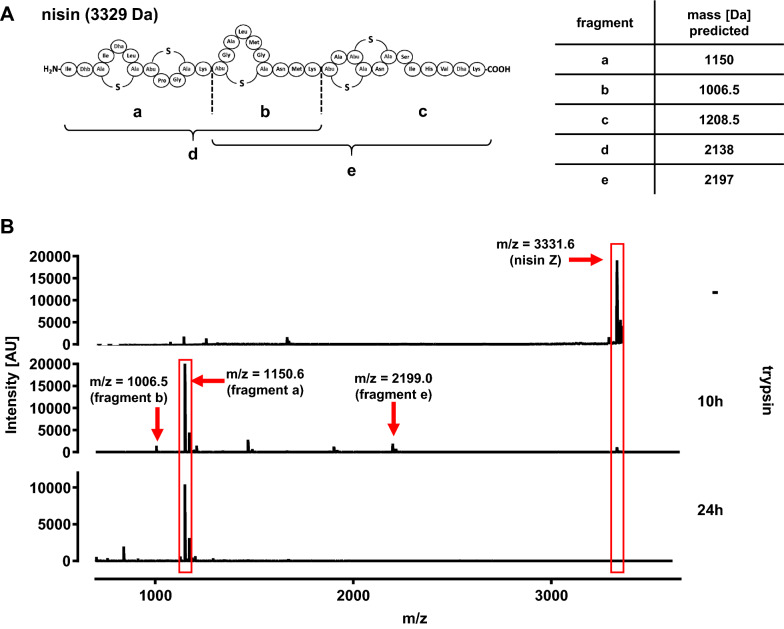
MS analysis of trypsin-treated nisin Z samples. **A** Schematic representation of mature nisin Z. Potential trypsin cleavage sites are indicated by dashed lines and the possible nisin Z fragments that result from complete or partial cleavage and their corresponding molecular mass (in Da) are indicated by different letters (a–e). **B** Mass spectrometry of untreated nisin Z (−) or after treatment for 10 and 24 h with trypsin. Peaks corresponding to mature active nisin Z or fragments predicted to result from complete or partial cleavage by trypsin are highlighted with red boxes and arrows and their mass/charge ratios are indicated

In natural producers such as *L. lactis* B1629, prenisin is activated to nisin by the specific membrane anchored protease NisP [20, 22], which cleaves the prepeptide at the arginine residue in position 23 removing the leader peptide (Additional file 1: Fig. S3C). In line with previous studies [21, 22, 57, 58], we achieved activation of prenisin using the serine protease trypsin (Figs. 2 and 3), which cleaves proteins non-specifically at arginine and lysine residues [59]. However, nisin Z contains two internal lysin residues that also may serve as a substrate for trypsin (Fig. 4A). In fact, MS analyses revealed several of the predicted degradation products when commercial nisin Z was incubated with trypsin for 10 h and 24 h (Fig. 4B). Moreover, only a very weak signal corresponding to the m/z of a nisin standard (m/z = 3331.6) after 10 h of trypsinization and this signal was completely lost after 24 h of treatment.

**Fig. 5:**
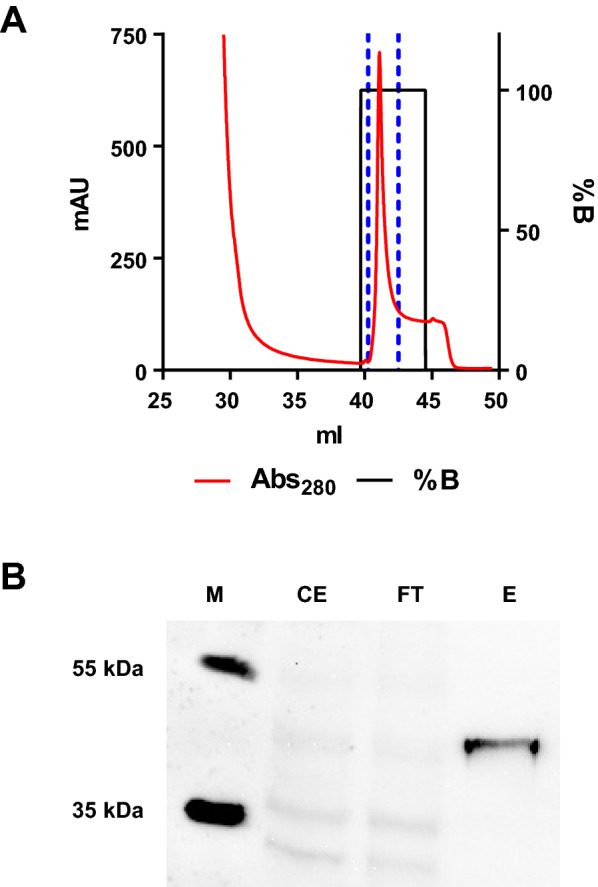
Purification of a His_6_-tagged soluble variant of NisP. **A** Immobilized metal affinity chromatography (IMAC) of crude extracts of *E. coli* BL21/pEKEx-*snisP*-His_6_. Indicated is absorbance at 214 nm (red, left y-axis) and % of elution buffer (%B, black, right y-axis) over the elution volume. **B** Western blot analysis after SDS-PAGE using α-His_6_ antibody. Samples taken at different steps of the purification of sNisP-His_6_. CE: crude extract; FT: flowthrough of IMAC; E: eluate of IMAC peak fraction; M: molecular weight marker

To improve specificity of the activation step and increase product yield, we adopted a previously described approach employing a soluble NisP protease (sNisP) [21, 22]. An *E. coli* BL21 derivative harboring pEKEx-*snisP*-His_6_ for expression of a His-tagged version of sNisP was cloned and sNisP-His_6_ was purified form crude extract of this strain by immobilized metal affinity chromatography (IMAC; Fig. 5A). Analysis of the peak fraction by SDS-PAGE and following Western blot indicated the presence of a His_6_-tagged protein migrating around the size expected for sNisPHis_6_ (42 kDa; Fig. 5B).

**Fig. 6:**
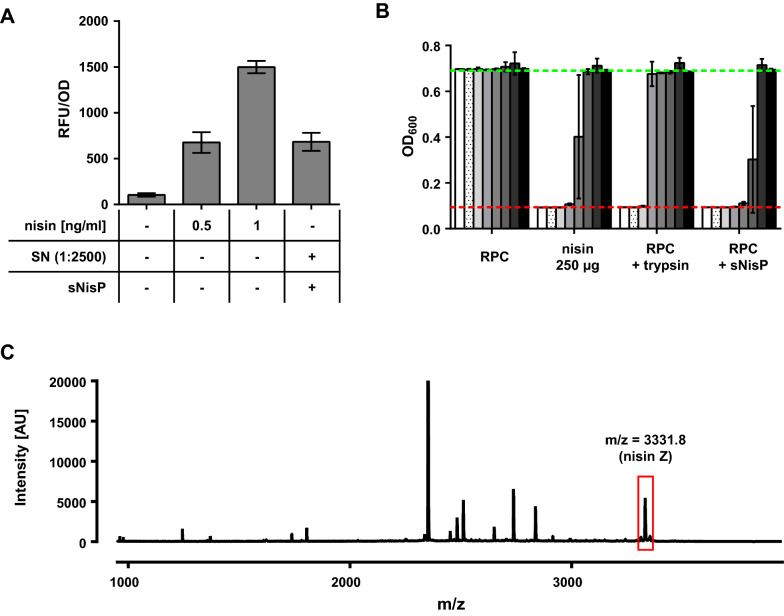
Detection of active nisin in SN of *C. glutamicum* CR099/pXMJ-*nisZBTC*^*Cg*^ or concentrated RP peak fractions activated with sNisP-His_6_. **A** RFU/OD of *L. lactis* NZ9000/pNZ-P_*nis*_*-mcherry*^*Ll*^ grown o/N in the presence of supernatants (SN) of *C. glutamicum* CR099/pXMJ-*nisZBTC*^*Cg*^ or nisin (0.5 or 1 ng/ml). Prior to assays, supernatants were activated by incubation with the sNisP-His_6_ preparation shown in Fig. 5B for 3.5 h (sNisP) and diluted 1:2500. **B** Inhibition of growth (OD_600_) of *L. lactis* IL1403 by serial twofold dilutions concentrated RP peak fraction (RPC) or nisin Z (starting concentration of the standard solution 250 µg/ml). Where indicated, the RP peak fraction was activated with either trypsin (0.5 mg/ml) or sNisP-His_6_ for 3.5 h. All values are mean ± SD of n = 3 independent cultures. As negative controls untreated SN or RP were included. The broken red and green lines indicate OD_600_ of the positive (i.e. complete inhibition of growth) or negative (i.e. sterile medium) controls, respectively. **C** Mass spectrometry of sNisP-activated SN analysed in (**A**, **B**). The peak corresponding to mature, active nisin Z are highlighted with a red box

This sNisP-His_6_ preparation was subsequently used to activate prenisin in supernatants of *C. glutamicum* CR099/pXMJ-*nisZBTC*^*Cg*^. When activated with sNisP-His_6_, supernatants diluted 1:2500 induced fluorescence of the mCherry biosensor comparable to 0.5 ng/ml nisin (Fig. 6A) indicating presence of at least 1.25 µg/ml of active nisin. Additionally, the sNisP-His_6_ preparation of the IMAC peak fraction was concentrated by molecular weight cutoff filtration (cut-off 30 kDa) and used to activate prenisin in a concentrated RP peak fraction. As a control, the same concentrated RP peak fraction was activated with trypsin. Two-fold serial dilutions of these samples were investigated for activity using a growth-dependent assay with the nisin sensitive *L. lactis* strain IL1403 [60] as indicator strain (Fig. 6B). The results indicate that samples activated with sNisP-His_6_ contained about two-fold higher levels of active nisin than the standard (250 µg/ml), i.e. approx. 500 µg/ml of active nisin Z. By contrast, activation with trypsin yielded only half the activity of the standard solution, i.e. approx. 125 µg/ml. This suggests that the protocol for activation with sNisP-His_6_ yields four-fold higher levels of active nisin than trypsin activation. Additionally, MS-analyses of prenisin samples from RP purification activated by sNisP-His_6_ were performed (Fig. 6C). A signal with a mass/charge ratio (m/z) of 3331.1 was detected, which is in good agreement with the m/z (3330.4) of the mature nisin Z reported in a previous study [61]. Despite a number of additional peaks of unknown origin, the MS data indicated that sNisP-His_6_-dependent activation did not result in non-specific nisin degradation products as observed for trypsin treatment (Fig. 4B).

## Discussion

At present, nisin is the only antimicrobial peptide approved as a food preservative by the FDA and EFSA [33, 62]. The global market for nisin reached 443 million USD in 2020 and is expected to increase to over 550 million USD by the year 2025 (marketsandmarkets.com). At the same time, the world is facing a dramatic increase in infections with antibiotic resistant bacteria and resistance to last resort antibiotics are rather common than an exception [4]. Hence, antimicrobial peptides are discussed as alternatives to classical antibiotics [5]. To allow use of bacteriocins for medical applications and ensure cost-efficient supply for other purposes, improved biotechnological production processes are needed. Production of nisin and other bacteriocins is currently performed with natural producer strains in batch fermentations on milk- or whey-based substrates [34, 36, 63]. These processes are well established and yield a product that is approved for use as food preservative and marketed world-wide. The complex media components of these substrates are, however, a major drawback for further downstream processing and purification steps [35] and limit the potential of nisin (and other bacteriocins) for clinical applications. Moreover, regulation of bacteriocin biosynthesis by feedback inhibition in natural producer strains results in low product yields [64]. An alternative to overcome these drawbacks may be transfer of nisin biosynthesis to a heterologous production host that can be cultivated to high cell densities on cheap, simple and well-defined media.

Heterologous production in *E. coli* was demonstrated for several lanthipeptides including nisin [65]. Similarly, biosynthesis of nisin and other lanthipeptides was transferred to *Bacillus subtilis* as expression host [66–68]. The present study was aimed at implementing a process for production of fully active nisin using *C. glutamicum*. This organism has several advantages: it has “generally regarded as safe” status, is widely used in biotechnology, can be cultivated to very high cell densities, is genetically well accessible, and several genome-reduced chassis strains are available [69–71]. Recently, we successfully demonstrated heterologous production of the class IIa bacteriocin pediocin PA-1 with *C. glutamicum* [45]. *C. glutamicum* lacks the receptor for pediocin, i.e. a mannose-PTS, and, consequently, is resistant to relevant concentrations of pediocin [45]. Nisin targets the universal cell wall precursor lipid II and hence a wide range of Gram-positive bacteria [26, 72]. It is thus not surprising that growth of *C. glutamicum* is inhibited by concentrations by far lower than the inhibitory concentrations of pediocin [45] making heterologous production of active nisin with *C. glutamicum* more difficult. We have thus tested various approaches to increase the resistance of *C. glutamicum* to nisin. These approaches included expression of (i) immunity proteins or (ii) ABC transporters known to confer nisin resistance in other organisms or their homologues in *C. glutamicum*, (iii) enzymes for alanylation or lysinylation of the cell envelope to introduce positive charges, and (iv) deletion of genes for porins of the outer membrane [47]. None of these approaches on their own yielded a substantial increase in resistance. Our results show that by combining, deletion of three porins, expression of an ABC transporter of *S. aureus* and addition of CaCl_2_, resistance of *C. glutamicum* to nisin was increased by a factor of 8. i.e. to around 5 µg/ml (Fig. 1B). Due to the low resistance of unmodified *C. glutamicum* to nisin and the rather marginal increase by various approaches, we decided to establish a two-step process with production of inactive prenisin using *C. glutamicum* and activation to nisin in a second downstream step.

Growth-dependent and pHluorin-dependent assays used and described here or elsewhere have a minimum detection limit in the low µg/ml range depending on the sensitivity of the sensor bacteria used [50, 73]. To establish a more sensitive and specific method to detect active nisin, we made use of the nisin autoregulation system of *L. lactis* [55]. An *mcherry* gene was fused to the promotor upstream of *nisZ* as reported previously [74], cloned into pNZ44 and introduced into *L. lactis* NZ9000 harboring the *nisRK* two-component system for sensing of mature nisin and regulation of the *nisZ* promoter. The obtained reporter stain showed strictly nisin-dependent mCherry florescence and had a limit of detection < 0.25 ng/ml of active nisin (Additional file 1: Fig. S2).

The minimal requirements for production of fully modified prenisin are the genes coding for the core peptide (*nisZ*) and for the modification and transport machinery (*nisBCT*), respectively [17]. In native producer strains, prenisin is cleaved by the membrane-anchored protease NisP [18] but presence of the arginine residue at position 23 allows cleavage and activation by trypsin [21, 22, 57]. For production of prenisin Z using *C. glutamicum*, a synthetic operon consisting of the genes *nisZBTC* codon-optimized for *C. glutamicum* each equipped with a strong ribosomal binding site was cloned under the IPTG-inducible P_tac_ promoter into pXMJ19. A recombinant *C. glutamicum* strain carrying this plasmid produced a compound that, after treatment with trypsin, was able to induce mCherry fluorescence in a highly specific nisin biosensor (Figs. 2 and 3). The purified compound had an m/z of 5666.7, which is in good agreement with the m/z of prenisin Z (5663). Of note, this compound was found in the supernatant of the recombinant producer. In a similar approach using expression of *nisABC* in *E. coli* active nisin was only obtained after purification of prenisin from crude extracts and activation by trypsin [65]. This clearly indicates functionality of the complete nisin biosynthesis, modification and export machinery in *C. glutamicum*, and successful production of completely modified prenisin Z that can be activated to mature nisin. Heterologous production of nisin (and other lantibiotics) in a two-step approach with downstream activation of the prepeptide has also been demonstrated with *B. subtilis* [66, 67]. However, in these studies successful production was demonstrated qualitatively and no quantification of the peptides or their bioactivity was performed. Thus, it is difficult to compare efficacy of (pre)nisin production with *B. subtilis* and *C. glutamicum* based on data available in literature.

Studies on heterologous production of (pre)nisin in *B. subtilis* made use of strains carrying an endogenous gene cluster for the lantibiotic subtilin [66, 67]. The recombinant producer strains were generated by replacing the gene for subtilin with a gene for prenisin plus other modification of the promoters. So, these strains produced prenisin with the biosynthetic machinery of subtilin. *C. glutamicum* lacks endogenous gene clusters for production of lanthipeptides and was modified to express the nisin biosynthetic machinery. This involved expression of membrane proteins for posttranslational modification and export. To our knowledge, this represents the first report on recombinant production of a posttranslationally modified peptide with heterologous expression of a complete set of enzymes for modification and transport.

It is hypothesized that trypsin only cleaves off the signal peptide and the presence of (methyl-)lanthionine rings prevents further proteolytic degradation at two lysin residues, i.e. potential trypsin cleavage sites, in the mature nisin molecule [75]. By contrast our MS data suggest unspecific cleavage of nisin at these lysin residues at least at longer incubation times (Fig. 4B). To increase specificity of prenisin activation, we adopted a strategy to produce a soluble version of the nisin-specific protease NisP as described previously [21, 22]. Following recombinant expression of sNisP-His_6_ in *E. coli* BL21 and purification by IMAC, prenisin was successfully activated in culture supernatants of *C. glutamicum* CR099/pXMJ-*nisZBTC*^*Cg*^ by adding sNisP-His_6_.

Based on semiquantitative determination using a standard solution of commercial nisin, at least 1.25 µg/ml were present in sNisP-activated culture supernatants. This is comparable to or slightly above levels reported for homologous production with *L. lactis* strains [58, 76]. Also, nisin activity was clearly improved compared to activation with trypsin (Fig. 6). Based on semiquantitative determination using a standard solution of commercial nisin, activity equivalent to approx. 500 µg/ml were obtained when RP-purified prenisin was activated with sNisP-His_6_. Moreover, this is about 20-fold higher than nisin levels obtained with *E. coli* [65].

## Conclusions

In summary, our data demonstrates successful establishment of a two-step approach with recombinant production of prenisin using *C. glutamicum* and downstream activation using a soluble NisP protease. Thus, our results demonstrate that *C. glutamicum* may be used as heterologous production host not only for the non-modified class IIa bacteriocin pediocin [45] but also the fully modified lantibiotic nisin. Moreover, our approach may also be used to produce other lantibiotics by simply replacing the *nisZ* gene in the expression vector because the nisin modification and export machinery is promiscuous to other lanthipeptides as demonstrated in *L. lactis* [77]. Together with purification protocols e.g. by RP chromatography and downstream activation, this may be an interesting alternative for production of bacteriocins. As our experiments were carried out in rather simple batch cultivations in shake flasks, transfer to fed-batch or continuous fermentation processes may help to increase product yield and productivity. Moreover, *C. glutamicum* may be engineered to utilize a wide range of substrates [38] offering the possibility of production on sustainable substrates. Admittedly, the described two-step process yields a product derived from genetically modified organisms. Further studies are needed to establish production and purification processes that comply with good manufacturing practices required for clinical applications.

## Methods

### Strains and growth conditions

Strains and plasmids used in this study are listed in Additional file 1: Table S1. Bacteria were cultivated in 2xTY complex medium (*C. glutamicum* and *E. coli*) or GM17 medium (*L. lactis*) with constant agitation (130 rpm) at 30 °C (*C. glutamicum* and *L. lactis*) or 37 °C (*E. coli*). For heterologous production of prenisin with *C. glutamicum*, 5 ml reaction tubes were inoculated from a single colony and incubated overnight (o/N). The next morning, cultures were transferred to 50 ml 2xTY medium containing 0.05 mM isopropyl-β-d-thiogalactoside (IPTG) in baffled Erlenmeyer flasks for further 8 h of cultivation. Then, the complete 50 ml cell culture was transferred to 2 l baffled Erlenmeyer flask containing 450 ml 2xTY, glucose and IPTG were added to a final concentration of 2% (w/v) and 0.2 mM, respectively. Cells were then cultivated o/N at 30 °C with constant agitation (100 rpm).

For growth experiments in 50 ml 2xTY medium main cultures were inoculated from o/N precultivated cells to an optical density (OD_600_) of 1.2 and cultivated for 48 h in 500 ml baffled Erlenmeyer flasks. Two hours after inoculation, prenisin synthesis was induced by 2% (w/v) glucose and 0.2 mM IPTG.

For selection either 12.5 µg/ml chloramphenicol (Cm; for pXMJ-*nisZBTC*^*Cg*^) or 50 µg/ml kanamycin (Kan; for pEKEx-*nisZBTC*^*Cg*^ or pEKEx-*snisP*-His_6_) was added where appropriate.

### Cloning procedures

Molecular cloning procedures were performed using standard reagents according to the manufacturer’s instructions. The prenisin biosynthesis genes *nisZ*, *nisB*, *nisC* and *nisT* were codon-optimized for expression in *C. glutamicum* obtained as synthetic DNA fragments from a commercial service provider (Eurofins Genomics). PCR reactions were performed in a C100 thermocycler (Bio-Rad Laboratories, Munich, Germany), nucleotides were purchased from Bio-Budget (Krefeld, Germany). All primer and gene sequences are listed in Additional file 1: Table S2. Original gene sequences were extracted from the genome sequence of the nisin Z producer strain *L. lactis* B1629 isolated from fermented purple aubergine (collection of D.B. Diep, Laboratory of Microbial Gene Technology, Norwegian University of Life Sciences). For cloning of plasmids for production of prenisin, the *nisZ*^*Cg*^ gene was excised from its cloning vector pEX-K168 by restrictions enzymes *Pst*I and *Sal*I and ligated into the shuttle vector pEKEx2 linearized by the same enzymes using T4-DNA ligase (Thermo Scientific). The resulting vector pEKEx-*nisZ*^*Cg*^ was used as backbone for a Gibson Assembly [78] approach to introduce further genes. The *nisB*^*Cg*^ gene was amplified via PCR using Q5 high fidelity polymerase (New England Biolabs) and appropriate primers creating overlapping regions with additional ribosome binding sites (RBS: 5ʹ-AAGGAGTTTTC-3ʹ) and restriction sites. The amplified *nisB*^*Cg*^ fragment was fused via Gibson Assembly into the backbone pEKEx-*nisZ*^*Cg*^ linearized by *Sal*I. The resulting plasmid pEKEx-*nisZB*^*Cg*^ was then again linearized by *Sal*I and fused with the PCR-amplified genes *nisT*^*Cg*^ and *nisC*^*Cg*^ in a second Gibson Assembly step yielding pEKEx-*nisZBTC*^*Cg*^. Finally, pEKEx-*nisZBTC*^*Cg*^ and empty vector pXMJ19 were digested with *Sal*I/*Sac*I and the *nisZBTC*^*Cg*^ fragment was inserted into the pXMJ19 backbone by T4 DNA ligation yielding pXMJ-*nisZBTC*^*Cg*^. Following cloning in *E. coli* DH5α, pXMJ-*nisZBTC*^*Cg*^ was transformed into *C. glutamicum* CR099 by electroporation as described previously [79].

For construction of a fluorescent nisin sensor plasmid the nisin-inducible promoter upstream of *nisZ* (P_*nis*_) was PCR amplified from *L. lactis* spp.* lactis* B1629 genomic DNA. Additionally, the gene coding for the red-fluorescent protein mCherry was obtained as a synthetic DNA fragment codon-optimized for expression in *L. lactis* spp. *cremoris* (Eurofins Genomics; Additional file 1: Table S2) and also amplified via PCR. Both PCRs were performed using primers for generation of overlapping sequences for subsequent Gibson Assembly. The pNZ44 vector [80] was linearized by restriction enzymes *Bgl*II and *Nco*I thereby removing the p44 promotor. The P_*nis*_ and *mcherry*^*Ll*^ fragments were fused to the linearized vector via Gibson Assembly resulting in the sensor plasmid pNZ-P_*nis*_*-mcherry*^*Ll*^ (Additional file 1: Fig. S2), which was transformed into *L. lactis* NZ9000 by electroporation [81] to obtain the nisin sensor strain *L. lactis* NZ9000/pNZ-P_*nis*_*-mcherry*^*Ll*^.

A soluble variant of the nisin protease nisP (sNisP) lacking the C-terminal LPXTG sortase motive was constructed as described previously [21]. The gene region coding for sNisP was amplified using *L. lactis* B1629 genomic DNA and primers snisP_fwd and snisP-6xH_rv (Additional file 1: Table S2) adding a 6× histidine tag (sNisP-His_6_). The PCR product was fused to the *BamH*I-linearized pEKEx2 vector by Gibson Assembly yielding pEKEx-*snisP*-His_6_. Following cloning in *E. coli* DH5α the construct was transformed into *E. coli* BL21 for efficient expression of sNisP-His_6_.

The plasmid pPB-pHin2^*Cg*^ was constructed as follows. Plasmid pPBEx2 [52] was digested with *Pst*I and *Pvu*II to remove the P_*tac*_ promoter as well as the first 1044 bp of the *lacI* gene. A fragment containing 179 bp upstream of the *tuf* gene of *C. glutamicum*, i.e. the highly active constitutive *tuf* promoter [82] fused to a *pHluorin2* gene [49] codon-optimized for *C. glutamicum* was synthesized by a commercial service provider (Eurofins Genomics), obtained in the pEX-K168 cloning vector. The insert was cut out and ligated to the pPBEx2 backbone to yield pPB-pHin2^*Cg*^. All plasmids were verified for correct cloning by restriction analysis and Sanger sequencing prior to transformation in their final hosts.

### Assessment of membrane damage

For detection of membrane damage, the pHluorin assay described recently [50], which is based on the ratiometric pHluorin by Miesenböck et al*.* [83], was adapted for *C. glutamicum*. For this purpose, *C. glutamicum* ATCC 13032 was transformed with pPB-pHin2^*Cg*^. The sensor strain *C. glutamicum* ATCC 13032/pPB-pHin2^*Cg*^ was grown o/N in 5 ml BHI containing 50 µg/ml Kan. The next day, cells were harvested by centrifugation and resuspended in Listeria minimal buffer (LMB, pH 6.2) at an optical density at 600 nm (OD_600_) of 3. Two-fold serial dilutions of samples for analysis were prepared in a black 96-well microtiter plates (Sarsted, Nümbrecht, DE) with a final volume of 100 µl in each well. Then, 100 µl of the sensor strain suspension was added and the plate was incubated at room temperature in the dark for 30 min. Readout was performed by measuring pHluorin2 fluorescence at 510 nm emission either across an excitation spectrum (350–490 nm) or with excitation at the distinct pHluorin2 maxima (400 and 470 nm) using an infinite M200 plate reader (Tecan, Männedorf, CH).

### Detection of activated pre-nisin using mCherry-based sensor bacteria

For analysis of nisin/prenisin standards or samples, 5 ml of GM17 medium supplemented with 10 µg/ml Cm was inoculated with a single colony of *L. lactis* NZ9000/pNZ-P_*nis*_*-mCherry*^*Ll*^ and grown for 8 h at 30 °C under continuous shaking (160 rpm). Afterwards, 100 µl of the preculture were used to inoculated 2.5 ml of fresh GM17 (+ 10 µg/ml Cm) medium containing the respective (pre-)nisin sample in the indicated dilution. As reference, different nisin standards and sole medium was used. The cultures were cultivated o/N at 30 °C under constant agitation (130 rpm). For mCherry fluorescence measurements, cells were centrifuged (3200*g*; 8 min; RT) and resuspended in 500 µl saline (0.9% (w/v)). Afterwards, 100 µl of each cell suspension was transferred to a black 96× well microtiter plate (Sarstedt) for measurements in technical triplicates. Fluorescence levels were determined in an Infinite M200 microplate reader (Tecan) with an excitation wavelength set to 570 nm and recorded emission at 610 nm. The optical density of the remaining cell suspension was determined spectroscopically and used to normalize the obtained fluorescence values.

### Growth inhibition assay

If appropriate, antimicrobial activity of previously activated prenisin samples was assessed by a growth inhibition assay in a similar manner as described before [45] using the nisin-sensitive *L. lactis* IL1403 as a sensor strain. Bacteria were grown o/N at 30 °C in GM17 medium containing 10 µg/ml Cm and diluted 1:25 in fresh GM17 prior to the assay. 100 µl of serial twofold dilutions of samples (100 µl) or a nisin standard were mixed with 100 µl of indicator bacteria in sterile 96-well plates. The plates were incubated at 30 °C for 6 h and growth was monitored by measuring the OD_600_ in an Infinite M200 plate reader (Tecan). Based on nisin standards of known concentration the nisin levels in activated prenisin samples were determined in a semi-quantitative manner. Growth inhibition assays were also used to evaluate nisin resistance levels of different *C. glutamicum* strains as described previously [47].

### Purification of prenisin

Purification of prenisin from cell-free supernatants was adopted from a previously described method [84]. In brief, supernatant proteins were precipitated o/N at 4 °C by (NH_4_)_2_SO_4_ (final concentration: 50% (w/v)). The precipitate was collected by centrifugation (45 min, 10,000*g*, 4 °C), resuspended in HPLC-grade H_2_O (1/10 of the initial volume), and pH was adjusted to 3.9 using 2 M HCl. After a further centrifugation step (45 min, 10,000*g*, 4 °C) to remove insoluble particles, the supernatant was applied to a HiPrep SP FF 16/10 column (GE Healthcare Life Sciences) for subsequent CIEX chromatography. Equilibration of column was done with 20 mM sodium phosphate buffer at pH 3.9 followed by 5 column volumes (CVs) washing step using 20 mM sodium phosphate buffer at pH 6.9. Elution was carried out with a single step using 5 CVs of 20 mM sodium phosphate buffer at pH 6.9 with 2 M NaCl. The eluate was collected to 5 ml fractions. Peak-fractions were pooled and applied to a Resource RP chromatography column (GE Healthcare Life Sciences). Elution of prenisin was done by a two-step protocol: (1) 5 CV of 10% buffer B (85% (v/v) acetonitrile with 0.1% (v/v) trifluoroacetic (TFA) acid), (2) linear increase of buffer B to 100% over 20 CV (flow rate for both steps: 1.0 ml/min). Prenisin eluted at approx. 50% of buffer B. All purification steps were performed with the Äkta-Pure system (GE Healthcare Life Sciences).

### Purification a His-tagged, soluble NisP protease

A soluble variant of the nisin protease NisP carrying an 6×His-tag (sNisP-His_6_) for purification by immobilized metal affinity chromatography (IMAC) was produced using *E. coli* BL21/pEKEx2-*snisP*-His_6_. A single colony of this strain was used to inoculate 5 ml of 2xTY. This culture was incubated o/N at 37 °C and then transferred to 50 ml 2xTY containing 0.2 mM IPTG and incubated at 37 °C for 8 h. To produce sNisP-His_6_, 250 ml Terrific broth (TB) medium were inoculated with 10% (v/v) of the 50 ml preculture and incubated o/N at 30 °C in the presence of 1 mM IPTG. To prepare sNisP-His_6_, bacteria of 50 ml aliquots of the o/N culture were harvested by centrifugation and each resuspended in 30 ml IMAC binding buffer (20 mM sodium phosphate buffer, pH 7.4; 0.5 M NaCl; 20 mM imidazole). Bacteria were disrupted by four passages through French Press (SLM Instruments) at 1100 psi. After removal of cell debris (2 × 10,000 g; 30 min; 4 °C) the supernatant, i.e. crude cell extract, was applied to the HisTrap FF 1 ml column (GE Healthcare Life Sciences) using a 50 ml super-loop. Protein bound to the columns was eluted by step gradient with high imidazole buffer (20 mM sodium phosphate buffer, pH 7.4; 0.5 M NaCl; 300 mM imidazole). To remove imidazole, 500 µl of the eluate were applied to a 30 kDa cut-off filter (Carl Roth GmbH) and retained protein sample was resuspended in 100 µl activation buffer (50 mM MOPS, pH 6.8; 50 mM NaCl) and stored at − 20 °C until usage for prenisin activation.

### SDS-PAGE and Western blot analysis

To confirm presence of sNisP-His_6_ in different fractions from previous purification, a Western blot was performed. First, a sodium dodecyl sulphate polyacrylamide gel electrophoresis (SDS-PAGE) on a tris-glycine, 10% polyacrylamide gel (Biorad) loaded with 10 µl of each sample fraction was performed. 5 µl of PageRuler™ prestained protein ladder 10–180 kDa (Thermo Scientific) were used as molecular weight marker. After electrophoresis (constant 30 mA; 250 V), gels were transferred for western blotting following a semi-dry blotting procedure in a Trans-blot turbo system (Biorad) according to the manufacturer’s protocol. For specific detection of sNisP-His_6_, a mouse mAb targeted against the 6×His-tag (Invitrogen, Thermo Scientific; Cat# MA1-21315, diluted 1:5000 in TBST buffer) was used. As secondary antibody, HRP-conjugated anti-Mouse goat IgG (Sigma-Aldrich; Cat# 12-349, diluted 1:5000 in TBST) was used. Detection was performed using the SuperSignal™ West Femto Maximum Sensitivity Substrate and the iBright Imaging System (ThermoFisher Scientific, Dreieich, Germany).

### Prenisin activation

Inactive prenisin can be activated either by the natural nisin protease NisP or using trypsin [21, 84], which both cleave after the arginine residue at position 23 of the leader peptide. For trypsin activation, supernatants, precipitation or samples from different purification steps were incubated with 1/10 of a 5 mg/ml trypsin (Sigma Aldrich) solution for 3.5 h at 37 °C. Activation by sNisP-His_6_ was performed using concentrated (by 30 kDa cut-off filter) purified sNisP-His_6_ dissolved in activation buffer. The mix of prenisin and sNisP was incubated for the indicated time period in a prenisin:sNisP-His_6_ ratio of 10:1. Activated prenisin samples were directly used for further analyses or stored at − 20 °C. For activation of purified prenisin, RP fractions were concentrated by evaporation in a speed vacuum concentrator (Eppendorf) and resuspended in the same activation buffer as sNisP-His_6_.

### Nisin trypsinization experiments

For trypsin digestion, nisin Z was purified from supernatants of the *L. lactis* producer strain by ammonium sulfate precipitation, followed by CIEX and RP chromatography. The purified nisin solution, containing 87% of nisin, was divided into three aliquots and dried at 55 °C in a SpeedVac concentrator (SPD2010 Integrated SpeedVac, ThermoFisher Scientific, USA). The pellet samples were resuspended in equal volumes of Tris-HCl 50 mM buffer, pH 8.0, containing 0.2 mg/ml of trypsin from porcine pancreas (Sigma Aldrich). The positive control sample was resuspended in Tris-HCl 50 mM buffer, pH 8.0 without trypsin. For partial digestion the nisin-trypsin mixture was incubated for 10 h at 37 °C. For total digestion the mixture was incubated for 24 h at 37 °C. Positive control sample was also incubated for 24 h at 37 °C.

To confirm the nisin digestion, activity was measured using a growth-dependent microtiter plate assay with *L. lactis* IL1403 as indicator as described above. Additionally, 5 µl of each sample was desalted with C18 loaded pipette tips (Millipore) and applied on MALDI-TOF spectrometer.

### MALDI-TOF analysis

For verification and further analysis of (pre)-nisin MALDI-TOF was performed. RP elution samples were concentrated by evaporation in a speed vacuum concentrator (Eppendorf) and resuspended in activation buffer (50 mM MOPS, pH 6.8; 50 mM NaCl) to 1/10 of the initial volume. Concentrated prenisin RP samples were analysed either with or without activation by sNisP-His_6_ or trypsin MALDI-TOF spectra were recorded on an Ultraflex III MS (Bruker Daltonics) operated in reflection mode with delayed extraction. Ions of positive charge in the *m/z* range of 200 to 6000 were analysed using 25 kV acceleration voltage. The sample spectra were calibrated externally with a calibration standard, *m/z* range from 700 to 3100 (Bruker Daltonics, Bremen, Germany).

## Supplementary Information

**Additional file 1: Figure S1.** Generation and properties of a *C. glutamicum* biosensor for detection of membrane damage. **Figure S2.** Generation and properties of *L. lactis* NZ9000/pNZ-P_*nis*_-*mcherry*^*Ll*^ biosensor for specific detection of nisin. **Figure S3.** Genetic organization of natural nisin Z operon of *L. lactis* B1629 and structure of prenisin Z. **Table S1.** Bacterial strains and plasmids used in this study. **Table S2.** Oligonucleotide primers and synthetic gene sequences used in this study.

## Data Availability

All data generated or analysed during this study are included in this published article and its additional information files.

## Abbreviations

A: Ampere
ABC-transporter: ATP binding cassette transporter
ATCC: American Type Culture Collection
BHI: Brain heart infusion
CTAB: Cetyltrimethylammonium bromide
Cm: Chloramphenicol
CIEX: Cation ion exchange chromatography
CV: Column volume
°C: Degree celsius
Da: Dalton
ESFA: European Food Safety Authority
et al.: Et alii
g: Gram
h: Hour
IPTG: Isopropyl-β-d-thiogalactopyranoside
IMAC: Immobilized metal ion affinity chromatography
k: Kilo
LAB: Lactic acid bacteria
l: Liter
MALDI/TOF: Matrix-assisted laser desorption-ionization/time of flight
MS: Mass spectrometry
MIC: Minimal inhibitory concentration
m/z: Mass/charge
M: Molar
m: Milli
min: Minute
µ: Micro
n: Nano
nm: Nanometer
OD_600_: Optical density at 600 nm
o/N: Over night
%: Percent
RPC: Reversed-phase chromatography
RT: Room temperature
SN: Supernatant
SD: Standard deviation
SDS-PAGE: Sodium dodecyl sulphate-polyacrylamide gel electrophoresis
V: Volt
WHO: World Health Organization
WT: Wildtype
