# Correction to: Recombinant production of the lantibiotic nisin using *Corynebacterium glutamicum* in a two‑step process

**DOI:** 10.1186/s12934-022-01744-1

**Published:** 2022-02-17

**Authors:** Dominik Weixler, Max Berghoff, Kirill V. Ovchinnikov, Sebastian Reich, Oliver Goldbeck, Gerd M. Seibold, Christoph Wittmann, Nadav S. Bar, Bernhard J. Eikmanns, Dzung B. Diep, Christian U. Riedel

**Affiliations:** 1 Institute of Microbiology and Biotechnology, University of Ulm, Albert-Ein-stein-Allee 11, 89081 Ulm, Germany; 2 Faculty of Chemistry, Biotechnology and Food Science, Norwegian University of Life Sciences, Ås, Norway; 3 Department of Biotechnology and Biomedicine, Technical University of Denmark, Lyngby, Denmark; 4 Institute of Systems Biotechnology, Saarland University, Saarbrücken, Germany; 5 Department of Chemical Engineering, Norwegian, University of Science and Technology, Trondheim, Norway

## Correction to: Microbial Cell Factories (2022) 21:11 https://doi.org/10.1186/s12934-022–01739-y

Following publication of the original article [1], the authors identified an error in Fig. 3d. The correct figure (Fig. 3) is given in this correction.

**Fig. 3:**
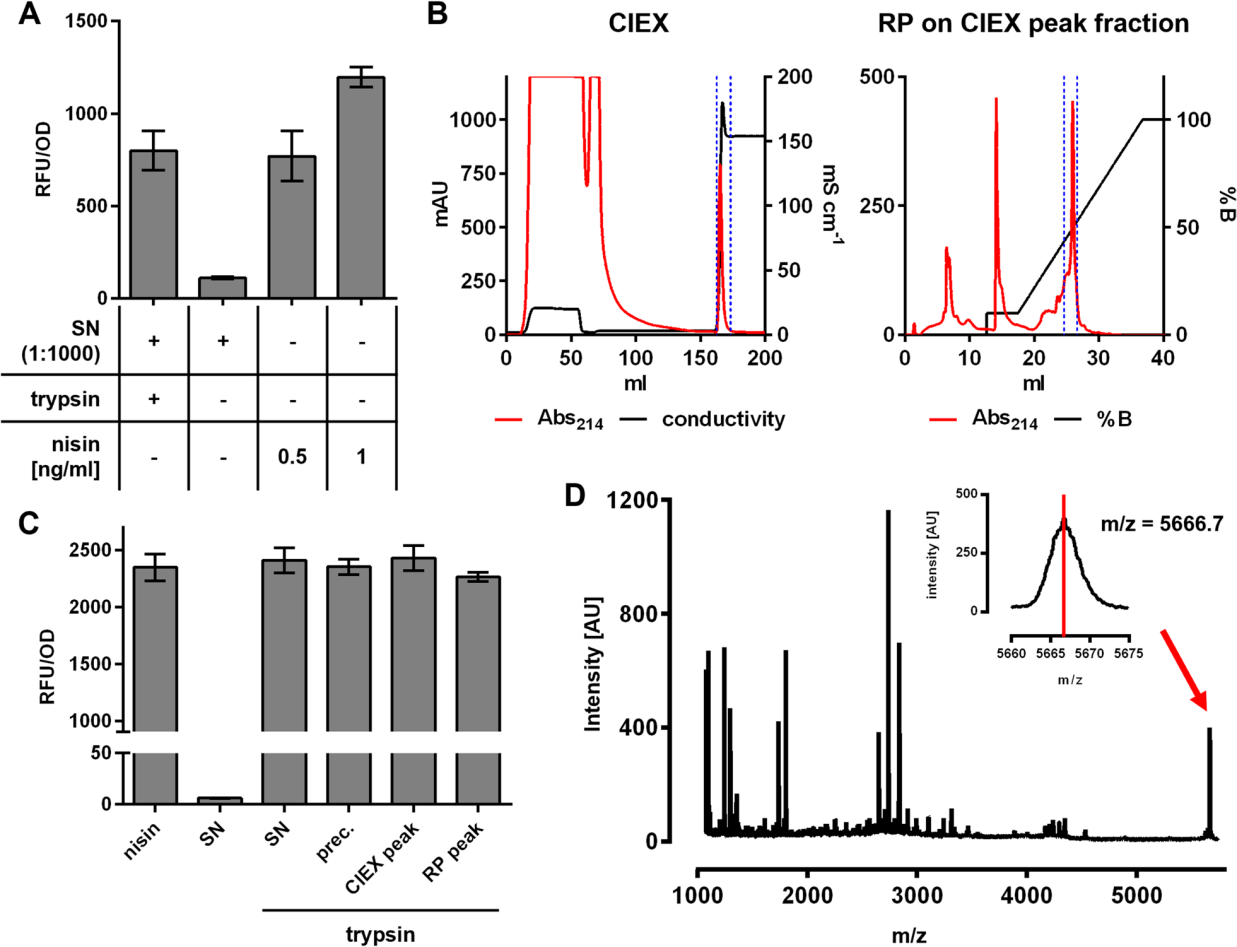
Purification and activation of prenisin produced by *C. glutamicum*. **A** Relative mCherry fluorescence normalized to OD (RFU/OD) of *L. lactis* NZ9000/pNZ-P*nis-mcherryLl* grown o/N in the presence of supernatants (SN) of *C. glutamicum* CR099/pXMJ-*nisZBTCCg*. The producer was grown o/N in 2xTY with 2% Glc and 0.2 mM IPTG. **B** Purification of ammonium sulphate-precipitated SN proteins by cation exchange (CIEX) and subsequent reverse phase (RP) chromatography on the CIEX peak fraction. Indicated is absorbance at 214 nm (red) and conductance (mS/cm; black, in CIEX) or % of elution buffer (%B, black, in RP) over the elution volume. Boundaries of the peak fractions further analysed are marked with blue broken lines. CRFU/OD of *L. lactis* NZ9000/pNZ-P*nis*-*mcherryLl* grown o/N in the presence of samples obtained at different steps during the purification of prenisin from SN shown in (**A**). prec: ammonium sulphate-precipitated SN proteins resuspended in pure H2O; CIEX and RP peak: peak fraction of the CIEX and RP chromatography. Prior to assays, samples were activated by incubation with trypsin (0.5 mg/ml for 3.5 h) and diluted 1:1000. As positive controls, the biosensor was grown in the presence of nisin Z at the indicated concentration. As negative controls, SN without trypsin treatment were included. **D** Mass spectrometry of the peak fraction obtained in RP chromatography in **B** with arbitrary peak intensity units (intensity [AU]) over mass/charge ratio (m/z). Values in **A** and **C** are mean ± SD of n = 3 independent cultures (**A**) or technical triplicates of one representative preparation (**C**)
