# Supplementary material for: Recombinant production of the lantibiotic nisin using Corynebacterium glutamicum in a two-step process
Source: Microb Cell Fact. 2022 Jan 15;21:11. doi: 10.1186/s12934-022-01739-y (PMC8760817; doi:10.1186/s12934-022-01739-y)
Supplement: Supplementary file 1 — Additional file 1: Figure S1. Generation and properties of a C. glutamicum biosensor for detection of membrane damage. Figure S2. Generation and properties of L. lactis NZ9000/pNZ-Pnis-mcherryLl biosensor for specific detection of nisin. Figure S3. Genetic organization of natural nisin Z operon of L. lactis B1629 and structure of prenisin Z. Table S1. Bacterial strains and plasmids used in this study. Table S2. Oligonucleotide primers and synthetic gene sequences used in this study. [file 12934_2022_1739_MOESM1_ESM.docx]

*Additional data*

Recombinant production of the lantibiotic nisin using *Corynebacterium glutamicum* in a two-step approach

Dominik Weixler^1^, Max Berghoff^1^, Kirill V. Ovchinnikov^2^, Sebastian Reich^1^, Oliver Goldbeck^1^, Gerd. M. Seibold^1,3^, Christoph Wittmann^4^, Nadav S. Bar^5^, Bernhard J. Eikmanns^1^, Dzung B. Diep^2^, and Christian U. Riedel^1,*^

^1^ Institute of Microbiology and Biotechnology, University of Ulm, Ulm, Germany

^2^ Faculty of Chemistry, Biotechnology and Food Science, Norwegian University of Life Sciences, Ås, Norway

^3^ Department of Biotechnology and Biomedicine, Technical University of Denmark, Lyngby, Denmark

^4^ Institute of Systems Biotechnology, Saarland University, Saarbrücken, Germany

^5^ Department of Chemical Engineering, Norwegian University of Science and Technology, Trondheim, Norway

* Corresponding author. E-mail: [christian.riedel@uni-ulm.de](mailto:christian.riedel@uni-ulm.de); Phone: +49 (0)731 50 24853. Institute of Microbiology and Biotechnology, University of Ulm, Albert-Einstein-Allee 11, 89081 Ulm, Germany

ORCID: 0000-0001-7134-7085


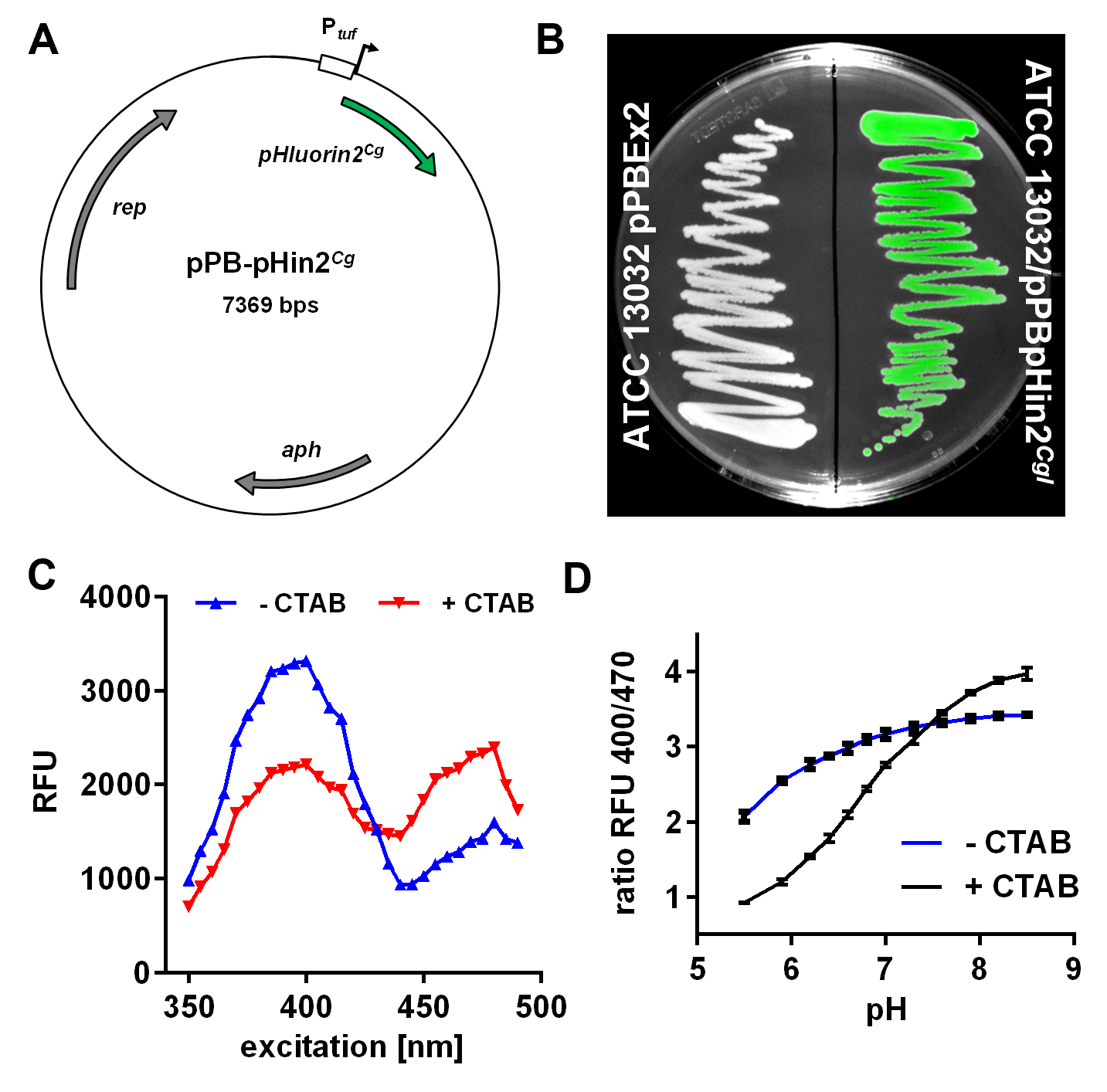


**Figure S1: Generation and properties of a *C. glutamicum* biosensor for detection of membrane damage.** **(A)** Plasmid map of pPB-pHin*^Cg^*, a pPBEx2-derived plasmid harboring a gene for the ratiometric pH-dependent protein pHluorin2 optimized for codon usage of *C. glutamicum* (*pHluorin2^Cg^*) driven by the strong, constitutive promoter P*_tuf_*. **(B)** Fluorescence imaging of the recombinant strain *C. glutamicum* ATCC 13032/pPB-pHin*^Cg^* and the empty vector control strain. Imaging was performed in an iBright^TM^ FL 1000 imaging system with filter settings for green fluorescence. **(C)** Relative fluorescence units at 520 nm (RFU) of *C. glutamicum* ATCC 13032/pPB-pHin*^Cg^* in LMB (pH 6.2) with (red) or without (blue) CTAB (0.001%) across a spectrum of excitation wavelengths (350-490 nm). **(D)** Ratios of fluorescence intensities (emission at 510 nm) with excitation at 400 or 480 nm (ratio RFU 400/470) of *C. glutamicum* ATCC 13032/pPB-pHin*^Cg^* in LMB adjusted to the indicated pH with (black) or without (blue) permeabilization by CTAB.


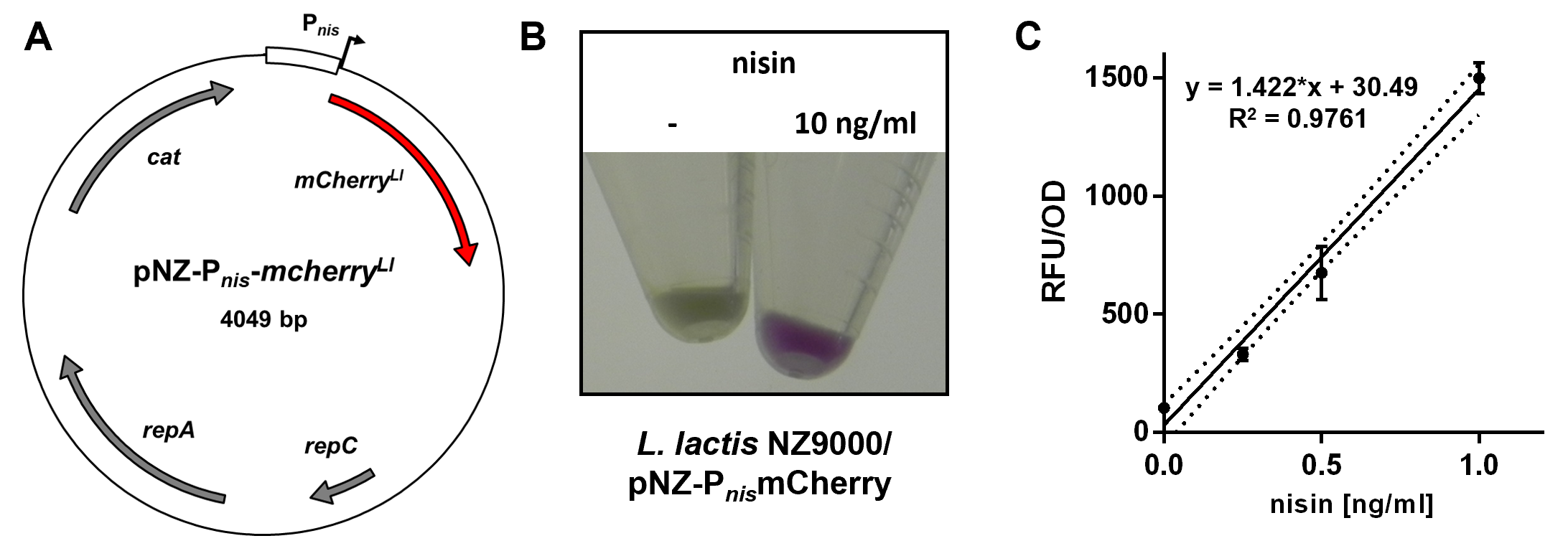


**Figure S2: Generation and properties of *L. lactis* NZ9000/pNZ-P*_nis_*-*mcherry^Ll^* biosensor for specific detection of nisin.** **(A)** Plasmid map of pNZ-P*_nis_mcherry^Ll^*, a pNZ44-derived plasmid harbouring a gene for the red fluorescent protein mCherry optimized for codon usage of *L. lactis* (*mcherry^Ll^*) driven by the strictly nisin-dependent P*_nis_* promotor. **(B)** Image of the recombinant strain *L. lactis* NZ9000/pNZ-P*_nis_*-*mcherry^Ll^* grown o/N with or without nisin Z (10 ng/ml). **(C)** OD_600_-normalized relative fluorescence (RFU/OD, excitation 570 nm, emission at 610 nm) of *L. lactis* NZ9000/pNZ-P*_nis_*-*mcherry^Ll^* following o/N growth in the presence of different concentrations of nisin Z (0, 0.25, 0.5, 1 ng/ml). Values are mean ± standard deviation (SD) of n = 3 independent cultures. The linear regression with SD and 95% confidence band were calculated and equation of the regression and Pearson’s coefficient of correlation (R^2^) are indicated.


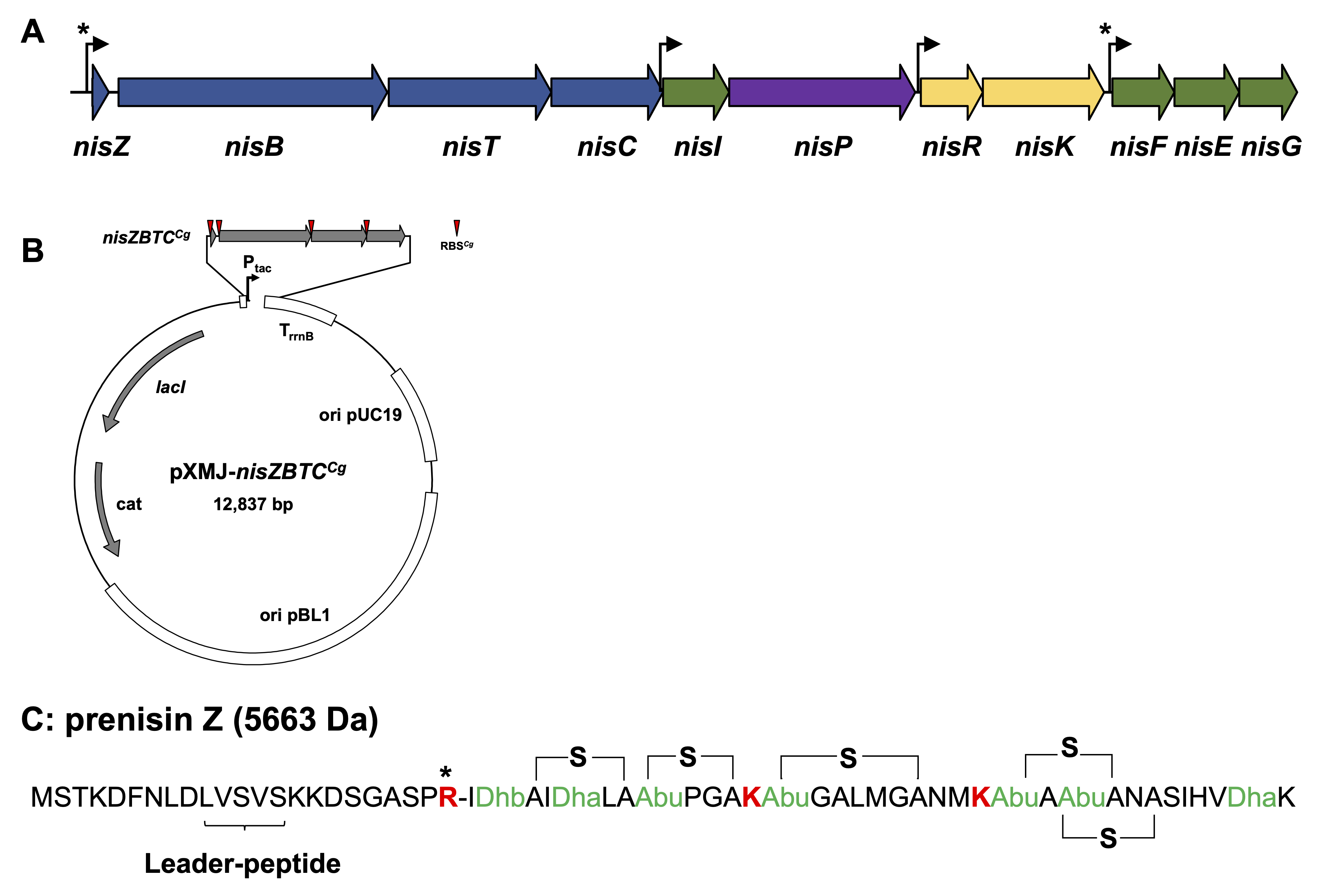


**Figure S3**: **Genetic organization of natural nisin Z operon of *L. lactis* B1629 and structure of prenisin Z.** (**A**). The genes of *L. lactis* B1629 for biosynthesis (blue), proteolytic activation (purple) of nisin Z, regulation (yellow) and resistance against the lantibiotic (green) are organized in an operon with promoters upstream of *nisZ*, *nisI*, *nisR* and *nisF*. Two of these promoters (upstream of *nisZ* and *nisF*, highlighted with an asterisk) are positively regulated by nisin via the *nisRK*-encoded two component regulatory system [1,2]. (**B**). Plasmid map of pXMJ-*nisZBTC^Cg^* for IPTG-inducible biosynthesis of prenisin in *C. glutamicum*. (**C**) Amino acid sequence with (methyl)lanthionine rings of fully modified prenisin Z. Unusual amino acids resulting from posttranslational modification steps are marked in green (Dhb: dehydrobutyrine; Dha: dehydroalanine; Abu: Aminobutyric acid). Cleavage site of the nisin-specific protease encoded by *nisP* is indicated by an asterisk. Potential trypsin cleavage sites (arginine and lysine residues) are highlighted in red.

**Table S1:** Bacterial strains and plasmids used in this study.

| **Strain** | **Relevant characteristics** | **Source** |
| --- | --- | --- |
| ***E. coli*** | | |
| DH5α | cloning host; F^-^ *endAI* *supE44* *thi-l* λ^-^ *recAl* *gyrA96* *relA*I *deo*R  Δ(*lacZY*A-*argF*)U169 Φ80d*lacZ*Δ*M15* *mcrA*  Δ(*mrr hsdRMS mcrBC*) | [3] |
| BL21 | *ompT*, *hsdSB* (rB^-^,mB^-^), *dcm*, *gal* (DE3) | [4] |
| ***Lactococcus lactis*** | | |
| IL-1403 | indicator strain | [5] |
| NZ9000 | indicator strain subsp. *cremoris* MG1363 *pepN*::*nisRnisK* | MoBiTec^®^ |
| B1629 | natural producer of nisin Z, isolated from fermented purple aubergine. | Dzung Diep (unpublished) |
| ***Corynebacterium glutamicum*** | | |
| CR099 | *C. glutamicum* ATCC 13032 ΔCGP1 ΔCGP2 ΔCGP3 ΔISCg1 ΔISCg2; cured of prophages CGP1, CGP2 and CGP3 and insertion elements ISCg1 and ISCg2 | [6] |
| ATCC 13032 | *C. glutamicum* type strain | ATCC |
| **Plasmid** | **Relevant characteristics** | **Source** |
| pNZ44 | shuttle vector, replicon for *E. coli* and a wide range of Gram-positive bacteria, high-copy number, Cm^R^ | [7] |
| pEKEx2 | *E. coli*/*C. glutamicum* shuttle vector; P*tacI*; *lacI*^q^; *oriC.g* from pBL1.; *oriE.c*. ColE1 from pUC18; Kan^r^. | [8] |
| pPBEx2 | pEKEx2 derivative containing correct *lacI*^q^ allele and lacking replicate sequences by deletion of nucleotides 930–1140 within DraI-SapI region. *EcoR*I site in MCS removed; Kan^R^. | [9] |
| pXMJ19 | *E. coli*/*C. glutamicum* shuttle vector; P*tacI*; *lacI*^q^; *oriC.g* from pBL1.; *oriE.c*. ColE1 from pUC18; Cm^r^. | [10] |
| pNZ-P*_nis_*-*mcherry^Ll^* | pNZ vector carrying *mcherry* gene codon-optimized for expression in *L. lactis* subsp*. cremoris* under control of nisin inducible promotor *P_nisZ_ from L. lactis* B1629 | this study |
| pEKEx-*nisZBTC^Cg^* | pEKEx2 derivative for IPTG-inducible expression of the synthetic prenisin Z operon *nisZBTC* | this study |
| pEKEx-*snisP*-His6 | pEKEx2 derivate for IPTG-inducible expression of a soluble variant of nisin protease NisP with C-terminal 6x histidine tag | this study |
| pPB-pHin2*^Cg^* | pPBEx2 derivate, P_tuf_-mediated constitutive expression of codon-optimized *pHluorin2* | this study |
| pXMJ-*nisZBTC^Cg^* | pXMJ19 derivative for IPTG-inducible expression of synthetic prenisin Z operon *nisZBTC* | this study |

**Table S2:** Oligonucleotide primers and synthetic gene sequences used in this study.

| **Primer** | **Sequence (5’ 🡪 3’)** | **Purpose** |
| --- | --- | --- |
| px2_fw  px2_rv | CACTCCCGTTCTGGATAATG  GCTACGGCGTTTCACTTCTG | Control primer flanking MCS pEKEx2, pPBEx2 and pXMJ19 |
| seq1 | CCTCCGAGCTGGAAGAAGTG | Sequencing primer binding in synthetic *nisZBTC^Cg^* operon |
| seq2 | CAGCTGCGAAATCGTATTCC |  |
| seq3 | TACCCTGGAACTGTCTGAAG |  |
| seq4 | TGCTGCTGATTGTGCCTGTG |  |
| seq5 | TCAAGAAGGCGTCTATCTAC |  |
| seq6 | AGCATCCCTGTCCGCACTTC |  |
| seq_rev | TGGTACGGTGGTAGCTGGTC |  |
| nisB^Cg^_fw  nisB^Cg^_rv | CATCCACGTTTCGAAGTAAGGTCGACAAGGAGTTTTCATGATCAAAAGCTCT  TCAAGCTTATTCATGAAAACTCCTTTTACTCGTCGTTATCTTCG | Amplification of *nisB^Cg^* for pEKEx2-*nisZBTC^Cg^* |
| nisT^Cg^_fw  nisT^Cg^_rv | CGACGAGTAAAAGGAGTTTTCATGAATAAGAAGAACATCAAACG  ATTCGAGCTCGGTACCCGGGGAGCTCTTATTTGCGTTTACCGCC | Amplification of *nisC^Cg^* for pEKEx2-*nisZBTC*^Cg^ |
| nisC^Cg^_fw  nisC^Cg^_rv | TATATGAAGTGACTCTAGAGAAGGAGTTTTCATGGATGAAGTGAAGGAG  CTTATTCATGAAAACTCCTTTTACTCGTCGTTATCTTCG | Amplification of *nisT^Cg^* for pEKEx2-*nisZBTC^Cg^* |
| pNZ_fw  pNZ_rv | AATAGCGACGGAGAGTTAGG  AACTAGTGGTACCGCATGCC | Control primer flanking MSC of pNZ |
| P_nisZ__fw  P_nisZ__rv | AGGTTTTTATATTACAGCTCCAATAGTCTTATAACTATACTGACAATAGAAAC  TACTCACCATCCATGGTTTGAGTGCCTCCTTATAATTTATTTTG | Amplification and assembly of P*_nisZ_* for pNZP*_nisZ_*_*mcherry^Ll^* |
| mch^Ll^_fw  mch^Ll^_rv | GGCACTCAAACCATGGATGGTGAGTAAAGGAGAG  TACCGCATGCCTGCAGTACCGGTACCTTACTTGTAAAGTTCATCCATC | Amplification and assembly of *mcherry^Ll^* for pNZP*_nisZ_*_*mcherry^Ll^* |
| snisP_fw  snisP-his_rv | CCTGCAGGTCGACTCTAGAGGTCGACAAGGAGTTTTCGTGAAAAAAATCCTAGGTTTC  ATTCGAGCTCGGTACCCGGGGAGCTCTTAATGGTGATGGTGATGGTGATCTGTATCTAAGCTAAAAGC | Amplification and assembly of *snisP-His6* for pEKEx2_snisP-His6 |
| pPTEx_fw | GCCTACAGCATCCAGGGTG | Forward sequencing primer for pPB-pHin2*^Cg^* |

**Table S2:** Continued

| **Gene**  **(size in bp)** | **Sequence (5’🡪 3’)** |
| --- | --- |
| *nisZ^Cg^*  (174) | ATGTCCACCAAAGACTTCAACCTCGATCTTGTGTCTGTCTCCAAGAAGGATTCTGGAGCTTCACCTCGCATTACCTCCATCTCCCGTGTACACCAGGCTGTAAGACTGGTGCCTTGATGGGCTGCAACATGAAAACCGCAACGTGCAATTGCAGCATCCACGTTTCGAAGTAA |
| *nisB^Cg^*  (2982) | TGATCAAAAGCTCTTTCAAGGCTCAGCCGTTTCTGGTGCGTAACACCATTCTGTCTCCGAACGACAAACGTTCTTTCACCGAATACACCCAGGTGATCGAAACCGTGAGCAAAAACAAAGTCTTTCTGGAACAGCTGCTGCTGGCTAACCCGAAACTGTATAATGTTATGCAGAAATACAACGCGGGTCTGCTGAAAAAGAAGCGCGTAAAGAAACTGTTCGAGAGCATCTATAAATACTATAAGCGCAGCTACCTGCGTAGCACCCCATTCGGTCTGTTCTCCGAAACCTCTATTGGCGTATTTTCTAAGTCTTCCCAGTATAAGCTGATGGGTAAGACTACCAAGGGCATCCGTCTGGACACTCAGTGGCTGATTCGCCTGGTACACAAAATGGAAGTTGACTTCTCCAAAAAACTGTCCTTCACCCGTAACAACGCCAATTATAAATTTGGCGACCGTGTGTTCCAGGTTTATACTATCAACTCCTCCGAGCTGGAAGAAGTGAACATTAAATATACCAACGTATATCAGATCATTTCCGAATTTTGCGAAAATGACTACCAGAAATACGAAGACATTTGCGAAACCGTTACCCTGTGCTACGGCGACGAATACCGTGAACTGTCTGGCCAGTATCTGGGCAGCCTGATTGTAAACCACTACCTGATCAGCAATCTGCAAAAAGACCTGCTGAGCGATTTCTCCTGGGATACCTTCCTGACCAAAGTGGAAGCGATTGACGAGGATAAAAAATATATCATCCCGCTGAAGAAGGTACAGAAATTCATTCAGGAGTACTCCGAAATCGAAATCGGTGAGGGTATTGAAAAACTGAAGGAAATTTACCAGGAGATGAGCCAGATTCTGGAAAACGACAACTACATTCAAATCGATCTGATTTCCGACTCCGAGATCAACTTTGATGTTAAACAGAAACAACAGCTGGAACACCTGGCGGAGTTTCTGGGTAACACTACCAAGTCCGTGCGCCGTACTTATCTGGATGATTACAAGGATAAATTTATCGAGAAATACGGCGTAGACCAGGAAGTTCAAATCACTGAACTGTTTGACAGCACCTTTGGCATCGGTGCGCCGTATAACTACAACCACCCGCGTAACGACTTCTACGAGTCCGAGCCGTCTACTCTGTATTACTCTGAAGAAGAACGTGAAAAATATCTGTCTATGTATGTGGAAGCAATTAAAAACCACAACGTTATTAATCTGGATGACCTGGAATCTCACTACCAAAAAATGGACCTGGAAAAAAAATCTGAACTGCAAGGCCTGGAGCTGTTTCTGAACCTGGCAAAAGAATATGAAAAAGATATTTTCATCCTGGGTGACATCGTAGGTAACAATAATCTGGGTGGTGCATCTGGTCGTTTCTCTGCACTGTCCCCGGAGCTGACCAGCTACCACCGTACCATCGTTGACTCTGTGGAACGCGAGAACGAAAACAAAGAAATCACCAGCTGCGAAATCGTATTCCTGCCTGAAAACATCCGTCATGCCAACGTTATGCACACCAGCATCATGCGTCGTAAAGTGCTGCCATTCTTCACGTCCACTTCCCACAACGAAGTTCAGCTGACTAACATTTACATTGGCATTGACGAAAAAGAAAAATTCTATGCGCGTGACATTAGCACCCAGGAAGTGCTGAAGTTCTACATCACTAGCATGTACAACAAAACCCTGTTTTCTAACGAACTGCGTTTTCTGTACGAAATCAGCCTGGACGATAAATTCGGTAACCTGCCGTGGGAACTGATCTATCGTGATTTCGACTATATCCCGCGCCTGGTTTTTGACGAAATCGTAATTAGCCCGGCCAAATGGAAAATTTGGGGTCGTAACGTAAACAACAAGATGACTATCCGTGAGCTGATTCAAAGCAAAGAGATTCCGAAAGAGTTCTATATTGTTAACGGTGACAACAAAGTGTATCTGTCCCAGGAAAACCCGCTGGATATGGAAATTCTGGAAAGCGCCATCAAAAAATCTAGCAAACGTAAAGACTTCATTGAACTGCAAGAATACTTCGAAGACGAAAACATCATCAACAAGGGTCAGAAAGGTCGTGTGGCGGACGTAGTGGTGCCATTCATCCGTACTCGTGCACTGGGTAACGAAGGTCGTGCTTTTATCCGTGAAAAGCGCGTGTCTGTTGAACGCCGTGAGAAACTGCCGTTTAACGAGTGGCTGTATCTGAAGCTGTACATCTCCATCAACCGTCAGAATGAGTTCCTGCTGAGCTATCTGCCGGACATTCAGAAAATCGTGGCAAACCTGGGTGGTAACCTGTTTTTTCTGCGTTACACCGACCCGAAGCCTCACATTCGTCTGCGCATCAAATGCAGCGACCTGTTTCTGGCGTACGGCTCTATTCTGGAAATCCTGAAACGTAGCCAGAAAAACCGTATTATGTCCACCTTCGATATTTCCATTTATGATCAGGAAGTCGAGCGTTATGGTGGTTTCGATACCCTGGAACTGTCTGAAGCAATCTTCTGCGCGGATTCTAAAATCATCCCGAACCTGCTGACCCTGATTAAAGATACCAACAACGACTGGAAAGTCGATGACGTTTCCATCCTGGTTAACTACCTGTACCTGAAGTGCTTTTTCCAGAACGATAACAAGAAGATTCTGAACTTCCTGAACCTGGTGTCTCCGAAAAAAGTAAAGGAAAACGTGAACGAAAAAATCGAACACTACCTGAAACTGCTGAAAGTGGACAACCTGGGCGATCAGATTTTCTACGACAAAAACTTCAAGGAACTGAAGCACGCAATCAAGAACCTGTTCCTGAAAATGATCGCGCAGGACTTTGAACTGCAAAAAGTATATTCTATCATCGACTCCATCATCCACGTTCATAACAACCGTCTGATCGGTATCGAGCGTGATAAAGAAAAACTGATCTATTACACCCTGCAACGTCTGTTCGTTAGCGAGGAATATATGAAGTGA |

**Table S2:** Continued

| **Gene**  **(size in bp)** | **Sequence (5’🡪 3’)** |
| --- | --- |
| *nisT^Cg^*  (1803) | ATGGATGAAGTGAAGGAGTTCACATCAAAGCAGTTCTTTAACACGTTGCTCACTTTTCCGTCAACCTTGAAGCTCATCTTCCAGCTGGAGAAACGTTATGCGATTTACCTTATTGTCCTGAACGCCATTACCGCATTTGTACCCCTGGCTTCTCTGTTTATCTACCAAGACCTTATTAACTCAGTTCTGGGCTCTGGTCGGCATCTTATCTATATCATCATCATTTACTTCATCGTCCAGGTGATCACTACGGTTCTGGGCCAACTTGAATCCTATGTTAGCGGTAAGTTCGACATGCGTCTTTCCTATTCCATCAATATGCGCTTGATGCGCACAACCTCCTCCCTGGAACTTTCGGATTATGAGCAGGCAGATATGTATAACATCATTGAGAAGGTCACTCAGGATAGCACCTATAAGCCGTTCCAGCTCTTCAATGCGATTATCGTGGTTCTGTCCTCGTTCATCTCACTGCTCTCCTCCTTGTTCTTCATTGGCACTTGGAACATCGGAGTTGCCATTCTGCTGCTGATTGTGCCTGTGCTGTCCCTCGTGCTGTTTCTGCGCGTAGGGCAGCTGGAGTTCCTTATCCAGTGGCAACGCGCTTCTAGCGAACGTGAAACCTGGTACATTGTGTACTTGCTTACCCATGACTTCAGCTTCAAGGAGATCAAGCTCAATAACATCTCGAACTACTTTATCCACAAATTTGGCAAGCTGAAGAAAGGCTTTATCAATCAGGATCTTGCCATCGCCCGCAAGAAAACCTACTTCAACATCTTTCTGGACTTCATCCTGAATTTGATCAACATCTTGACCATCTTCGCTATGATCCTGTCAGTCCGAGCAGGCAAATTGTTGATTGGGAATCTGGTTTCCCTCATTCAGGCGATTTCCAAGATTAACACCTACTCTCAGACGATGATTCAGAACATCTACATCATCTACAACACCTCCCTCTTCATGGAACAGTTGTTCGAATTCCTCAAACGCGAATCAGTGGTTCACAAGAAAATCGAGGATACAGAGATCTGCAATCAGGATATCGGAACGGTAAAAGTCATCAACCTGTCCTACGTCTACCCTAACTCCAATGCCTTTGCACTCAAGAACATCAACCTCTCTTTCGAGAAGGGTGAATTGGCGGCTATTGTGGGTAAGAACGGTTCTGGCAAATCGACCCTCGTTAAGATCATTAGCGGCCTCTATCAACCCACTATGGGCATTATTCAGTACGACAAGATGCGTTCCTCGCTCATGCCAGAGGAGTTTTACCAGAAGAACATCTCGGTGTTGTTCCAGGACTTCGTGAAATACGAGCTTACCATCCGCGAAAACATTGGTCTGAGCGACCTGTCCTCCCAGTGGGAAGATGAGAAAATCATCAAAGTCCTTGACAACCTCGGTCTTGACTTCCTCAAGACCAACAACCAGTACGTTCTGGACACCCAGCTGGGGAATTGGTTCCAAGAAGGACGCCAATTGAGCGGAGGTCAATGGCAGAAGATCGCACTGGCACGGACTTTCTTCAAGAAGGCGTCTATCTACATTCTGGACGAACCATCCGCTGCACTCGATCCAGTAGCAGAGAAGGAGATCTTCGACTACTTTGTCGCCCTTTCCGAAAACAATATCTCCATCTTCATTTCTCACAGCCTTAATGCTGCTCGCAAAGCTAACAAGATCGTCGTTATGAAGGATGGCCAAGTCGAAGATGTGGGATCTCACGATGTCCTGCTGCGACGTTGTCAGTACTACCAAGAACTGTACTATTCCGAGCAATACGAAGATAACGACGAGTAA |
| *nisC^Cg^*  (1245) | ATGAATAAGAAGAACATCAAACGGAACGTTGAAAAGATCATTGCTCAGTGGGATGAACGCACACGCAAGAACAAAGAAAACTTCGACTTTGGCGAACTGACGCTTTCCACCGGTTTGCCGGGAATTATCCTCATGCTTGCCGAGTTGAAGAACAAGGATAACAGCAAGATCTACCAGAAGAAGATTGACAACTATATCGAGTACATTGTCAGCAAACTGTCCACCTATGGTTTGTTGACCGGTTCGCTCTACTCTGGAGCTGCCGGAATCGCTCTGTCTATCCTCCACCTCCGTGAGGATGACGAGAAGTACAAGAATCTTCTGGATTCTCTGAACCGCTACATCGAGTACTTCGTTATCGAAAAGATCGAGGGCTTCAATCTGGAGAACATCACTCCACCCGACTACGATGTGATCGAAGGTCTGTCAGGGATTCTGTCATACTTGCTTCTCATCAACGATGAACAGTACGACGATCTCAAGATCCTGATCATCAACTTTCTGTCGAATCTCACCAAGGAGAACAAGGGTTTGATCTCGCTCTACATCAAAAGCGAGAATCAGATGAGCCAATCAGAATCGGAAATGTATCCTCTGGGTTGTCTGAACATGGGCCTTGCTCATGGCCTGGCTGGAGCAGGGTGCATTCTTGCATACGCGCACATTAAGGGGTATTCCAACGAAGCATCCCTGTCCGCACTTCAGAAAATCATCTTCATCTACGAAAAGTTCGAACTCGAACGCAAGAATCAGTTTCTGTGGAAAGACGGTCTGGTAGCCGATGAACTTAAGAAAGAGAAAGTGATTCGAGAAGCGTCCTTCATTCGTGATGCATGGTGCTATGGTGGCCCAGGCATTTCCTTGCTTTACCTCTATGGAGGCCTCGCGCTTGATAACGACTACTTCGTGGATAAAGCCGAGAAGATCCTGGAGTCTGCGATGCAACGCAAGCTGGGGATCGACTCCTACATCATCTGTCACGGCTACTCAGGCCTCATTGAGATTTGCTCCTTGTTCAAACGGTTGCTGAACACGAAGAAGTTCGACTCCTACATTGAGGAGTTCAACGTCAATTCCGAGCAAATCCTGGAAGAGTATGGCGATGAGTCTGGTACTGGCTTTCTGGAAGGCATCTCCGGATGCATTCTTGTCTTGTCCAAGTTTGAATACTCTATCAACTTCACCTATTGGCGTCAGGCACTGCTCCTGTTCGACGACTTTCTGAAAGGCGGTAAACGCAAATAA |

**Table S2:** Continued

| **Gene**  **(size in bp)** | **Sequence (5’🡪 3’)** |
| --- | --- |
| *mcherry^Ll^*  (711) | ATGGTGAGTAAAGGAGAGGAAGATAATATGGCGATTATAAAAGAGTTTATGCGCTTTAAGGTTCACATGGAAGGTAGTGTTAATGGGCATGAGTTTGAAATTGAAGGAGAAGGTGAAGGTCGTCCATATGAGGGAACTCAAACAGCTAAGTTGAAAGTTACCAAAGGAGGTCCTTTACCGTTTGCTTGGGACATTCTTTCACCCCAATTCATGTATGGGTCAAAAGCATATGTGAAACATCCTGCAGATATTCCGGATTATCTAAAATTAAGCTTTCCTGAAGGGTTTAAGTGGGAAAGAGTCATGAACTTTGAAGATGGTGGAGTAGTTACTGTAACTCAAGATTCATCTTTACAAGATGGCGAATTCATTTACAAAGTCAAATTACGTGGCACAAATTTTCCAAGTGATGGACCTGTTATGCAAAAGAAAACTATGGGATGGGAAGCATCTTCTGAACGAATGTATCCAGAAGATGGAGCCTTAAAAGGCGAAATCAAACAACGACTTAAATTGAAAGATGGTGGTCATTATGACGCTGAAGTGAAAACAACCTATAAAGCAAAGAAACCAGTTCAGTTACCAGGTGCCTACAATGTAAACATCAAATTGGACATTACGTCACACAATGAGGATTATACGATAGTTGAACAGTATGAAAGAGCTGAAGGTAGACATTCAACAGGAGGGATGGATGAACTTTACAAGTAA |
| P*_tuf_*-*pHluorin2^Cg^*  (940) | CGGGAAACCTGTCGTGCCAGCTGCCACAGGGTAGCTGGTAGTTTGAAAATCAACGCCGTTGCCCTTAGGATTCAGTAACTGGCACATTTTGTAATGCGCTAGATCTGTGTGCTCAGTCTTCCAGGCTGCTTATCACAGTGAAAGCAAAACCAATTCGTGGCTGCGAAAGTCGTAGCCACCACGAAGTCCAGGAGGACATACCATGTCCAAAGGTGAAGAGCTGTTCACTGGCGTTGTACCCATCTTGGTGGAGCTTGATGGTGACGTGAATGGCCATAAGTTCAGCGTATCTGGTGAAGGAGAAGGTGACGCCACCTATGGCAAACTCACCCTGAAGTTCATCTGTACCACTGGCAAACTGCCTGTTCCATGGCCAACCTTGGTGACTACGTTGTCCTACGGTGTCCAGTGCTTTTCGCGCTATCCAGACCACATGAAGCAGCATGACTTCTTCAAATCAGCAATGCCAGAAGGTTACGTTCAAGAACGGACCATCTTCTTTAAGGATGATGGCAACTATAAGACCCGTGCTGAAGTCAAGTTTGAGGGCGATACCCTGGTGAACCGCATTGAACTGAAGGGAATCGACTTCAAGGAGGATGGCAACATCCTCGGGCACAAACTCGAGTACAACTACAACGAGCATCTGGTGTACATTATGGCCGACAAACAGAAGAATGGCACAAAGGCGATCTTTCAGGTCCACCACAACATCGAAGATGGCTCTGTCCAACTCGCTGACCACTACCAGCAGAATACGCCCATTGGAGATGGTCCGGTTCTTCTGCCTGACAACCACTACCTTCACACCCAATCCGCACTGTCCAAGGACCCGAACGAAAAGCGAGATCACATGGTTCTCCTTGAGTTCGTCACAGCAGCGGGAATTACCCATGGGATGGATGAGCTGTACAAGTAACTGCAGGTCGACTCTAGAGGA |

**Additional References**

1. Kuipers OP, Beerthuyzen MM, De Ruyter PGGA, Luesink EJ, De Vos WM. Autoregulation of nisin biosynthesis in Lactococcus lactis by signal transduction. J Biol Chem. © 1995 ASBMB. Currently published by Elsevier Inc; originally published by American Society for Biochemistry and Molecular Biology.; 1995;270:27299–304.

2. de Ruyter PG, Kuipers OP, Beerthuyzen MM, van Alen-Boerrigter I, de Vos WM. Functional analysis of promoters in the nisin gene cluster of Lactococcus lactis. J Bacteriol. 1996;178:3434–9.

3. Hanahan D. Studies on transformation of Escherichia coli with plasmids. J Mol Biol. J Mol Biol; 1983;166:557–80.

4. Studier FW, Moffatt BA. Use of bacteriophage T7 RNA polymerase to direct selective high-level expression of cloned genes. J Mol Biol. 1986;189:113–30.

5. Bolotin A, Wincker P, Mauger S, Jaillon O, Malarme K, Weissenbach J, et al. The complete genome sequence of the lactic acid bacterium lactococcus lactis ssp. lactis IL1403. Genome Res. 2001;11:731–53.

6. Baumgart M, Unthan S, Rückert C, Sivalingam J, Grünberger A, Kalinowski J, et al. Construction of a Prophage-Free Variant of Corynebacterium glutamicum ATCC 13032 for Use as a Platform Strain for Basic Research and Industrial Biotechnology. Appl Environ Microbiol. 2013;79:6006–15.

7. McGrath S, Fitzgerald GF, Van Sinderen D. Improvement and optimization of two engineered phage resistance mechanisms in Lactococcus lactis. Appl Environ Microbiol. 2001;67:608–16.

8. Eikmanns BJ, Thum-Schmitz N, Eggeling L, Ludtke KU, Sahm H. Nucleotide sequence, expression and transcriptional analysis of the Corynebacterium glutamicum gltA gene encoding citrate synthase. Microbiology. Microbiology Society; 1994;140:1817–28.

9. Bakkes PJ, Ramp P, Bida A, Dohmen-Olma D, Bott M, Freudl R. Improved pEKEx2-derived expression vectors for tightly controlled production of recombinant proteins in Corynebacterium glutamicum. Plasmid. Academic Press Inc.; 2020;112.

10. Jakoby M, Ngouoto-Nkili C-E, Burkovski A. Construction and application of new Corynebacterium glutamicum vectors. Biotechnol Tech. 1999;13:437–41.
